# Mucosa-associated microbiota drives pathogenic functions in IBD-derived intestinal iNKT cells

**DOI:** 10.26508/lsa.201800229

**Published:** 2019-02-13

**Authors:** Claudia Burrello, Gabriella Pellegrino, Maria Rita Giuffrè, Giulia Lovati, Ilaria Magagna, Alice Bertocchi, Fulvia Milena Cribiù, Francesca Boggio, Fiorenzo Botti, Elena Trombetta, Laura Porretti, Antonio Di Sabatino, Maurizio Vecchi, Maria Rescigno, Flavio Caprioli, Federica Facciotti

**Affiliations:** 1 Department of Experimental Oncology, IEO, European Istitute of Oncology IRCCS, Milan, Italy; 2 Gastroenterology and Endoscopy Unit, Fondazione IRCCS Ca’ Granda, Ospedale Maggiore Policlinico, Milan, Italy; 3 Department of Pathophysiology and Transplantation, Università degli Studi di Milano, Milan, Italy; 4 Pathology Unit, Fondazione IRCCS Ca’ Granda, Ospedale Maggiore Policlinico, Milan, Italy; 5 General and Emergency Surgery Unit, Fondazione IRCCS Ca’ Granda Ospedale Maggiore Policlinico, Milan, Italy; 6 Flow Cytometry Service, Clinical Chemistry and Microbiology Laboratory Fondazione IRCCS Ca’ Granda Ospedale Maggiore Policlinico, Milan, Italy; 7 First Department of Internal Medicine, Fondazione IRCCS Policlinico San Matteo, Pavia, Italy; 8 Department of Biomedical Sciences, Humanitas University, Pieve Emanuele, Italy

## Abstract

Inflammatory bowel disease (IBD) pathogenesis has been linked to the aberrant activation of the Gut-associated lymphoid tissues against components of the intestinal microbiota. Although the contribution of CD4^+^ T helper cells to inflammatory processes is being increasingly acknowledged, the functional engagement of human invariant natural killer T (iNKT) cells is still poorly defined. Here, we evaluated the functional characteristics of intestinal iNKT cells during IBD pathogenesis and to exploit the role of mucosa-associated microbiota recognition in triggering iNKT cells’ pro-inflammatory responses in vivo. Lamina propria iNKT cells, isolated from surgical specimens of active ulcerative colitis and Crohn’s disease patients and non-IBD donors, were phenotypically and functionally analyzed ex vivo, and stable cell lines and clones were generated for in vitro functional assays. iNKT cells expressing a pro-inflammatory cytokine profile were enriched in the lamina propria of IBD patients, and their exposure to the mucosa-associated microbiota drives pro-inflammatory activation, inducing direct pathogenic activities against the epithelial barrier integrity. These observations suggest that iNKT cell pro-inflammatory functions may contribute to the fuelling of intestinal inflammation in IBD patients.

## Abstract (precis)

Pro-inflammatory iNKT cells are enriched in IBD patients’ lamina propria. Exposure to the mucosa-associated microbiota drives their activation, inducing pathogenic activities against the epithelium.

## Introduction

Crohn’s disease (CD) and ulcerative colitis (UC), known as inflammatory bowel diseases (IBDs), are chronic inflammatory disorders of the digestive tract (Kaser et al, 2010) occurring in genetically predisposed individuals as the result of an abnormal immune response of gut-associated lymphoid tissues (GALT) against components of the intestinal microbiota (Belkaid & Hand, 2014). Whereas conventional CD4^+^ Th cells have been shown to play a major role in orchestrating intestinal inflammatory responses (Caprioli et al, 2008), the contribution of other mucosal T cell populations in sustaining or controlling intestinal inflammation is still under investigation (Heller et al, 2002; Fuss et al, 2004; Biancheri et al, 2014; Burrello et al, 2018b).

Among unconventional lymphocytes, CD1d-restricted T cells are a heterogeneous population recognizing endogenous and bacterial lipid antigens (Behar & Porcelli, 2007; Tupin et al, 2007; Facciotti et al, 2012), a feature distinguishing them from peptide-specific major histocompatibility complex (MHC)-restricted T cells. Different subsets of CD1d-restricted T cells have been identified over the years (Engel et al, 2016), mostly differing for their TCR repertoire and their different function in defined immune responses. Type I invariant natural killer T (iNKT) cells, widely studied in mice and men, express a conserved αβ T cell receptor (TCR; Vα24-Jα18/Vβ11 in humans and Vα14-Jα18 in mice) together with NK surface receptors and manifest both adaptive and innate/cytotoxic functional properties (Bendelac et al, 2007). Conversely, type II NKT express diverse TCRs, react to non-self and self-lipid antigens, including sulfatide (Marrero et al, 2015), and have been described to play critical roles in in the regulation of immunity to pathogens and tumors and in autoimmune disorders (Dhodapkar & Kumar, 2017).

Although both NKT cell subsets are present in the intestinal lamina propria (LP) (Middendorp & Nieuwenhuis, 2009), their specific role in gut mucosal immunity and regulation of intestinal inflammation have been only partially elucidated (Biancheri et al, 2014). Whereas the pro-inflammatory role of type II NKT cells has been clearly demonstrated in human UC patients (Fuss et al, 2004, Fuss et al, 2014) and in the chemically induced oxazolone-driven experimental colitis (Heller et al, 2002; Iyer et al, 2018), the role of type I iNKT cells is still controversial. In fact, iNKT cells have been reported to either contribute to experimental intestinal inflammation (Kim & Chung, 2013; Burrello et al, 2018a) or protect mice from experimental colitis in murine models (Saubermann et al, 2000; Ueno et al, 2005). Moreover, their functions in human IBD are still largely unexplored.

Current evidences suggest that intestinal inflammation in IBD is driven by stimulation of GALT by a dysbiotic gut microbiome (Strober, 2013; Gevers et al, 2014; Shah et al, 2016). This, in turn, is favored by IBD-associated defects in intestinal barrier functions (Grivennikov et al, 2012; Kamada & Nunez, 2013; Strober, 2013; Michielan & D'inca, 2015), which promote bacterial translocation in the intestinal LP (Fava & Danese, 2011), thus favoring the aberrant activation of both innate and adaptive mucosal immune responses. At present, however, whether similar events contribute to confer pro-inflammatory functions to intestinal iNKT cells in IBD patients has not been elucidated. In this context, it is well known that iNKT cells become activated upon recognition of pathogenic bacteria during infections (Tupin et al, 2007). More recently, a reciprocal influence between iNKT cells and the commensal gut microbiota has been demonstrated (Middendorp & Nieuwenhuis, 2009; Wei et al, 2010; Olszak et al, 2012; Burrello et al, 2018a), and increasing evidences support the existence of mutual mechanisms of regulation between the intestinal microbiota and iNKT cells (Nieuwenhuis et al, 2009). During early neonatal and postnatal stages of development, commensal bacteria negatively shape iNKT cell repertoire through a CXCL16-dependent gradient (Olszak et al, 2012). In addition, under homeostatic conditions, CD1d-dependent lipid antigens isolated from the commensal *B. fragilis* directly influence iNKT cell proliferation and activation status (An et al, 2014).

In this study, ex vivo LP cells isolated form surgical specimens of IBD patients and non-IBD donors were analyzed to evaluate frequency and phenotype of human intestinal iNKT cells. The direct stimulatory potential of the mucosa-associated intestinal microbiota, the microbial ecology most directly in contact with GALT and which represents the first microbial encounter of the GALT upon epithelial barrier disruption, was additionally tested on human iNKT cells and in murine chronic models of colitis.

Our data demonstrate that human intestinal iNKT cells are made up by a complex population, which is phenotypically distinct between IBD and non-IBD patients. Recognition of mucosa-associated intestinal microbiota induces pro-inflammatory cytokine secretion by iNKT cells, promoting direct pathogenic activities against the gut epithelial barrier.

Altogether, these findings shed novel light on the contribution of human intestinal iNKT cells in IBD pathogenesis, suggesting similar functions between iNKT cells and conventional CD4^+^ T cells during intestinal inflammation.

## Results

### Intestinal iNKT cells from IBD patients express a distinct pattern of surface molecules and secrete pro-inflammatory cytokines

**Figure 1.**
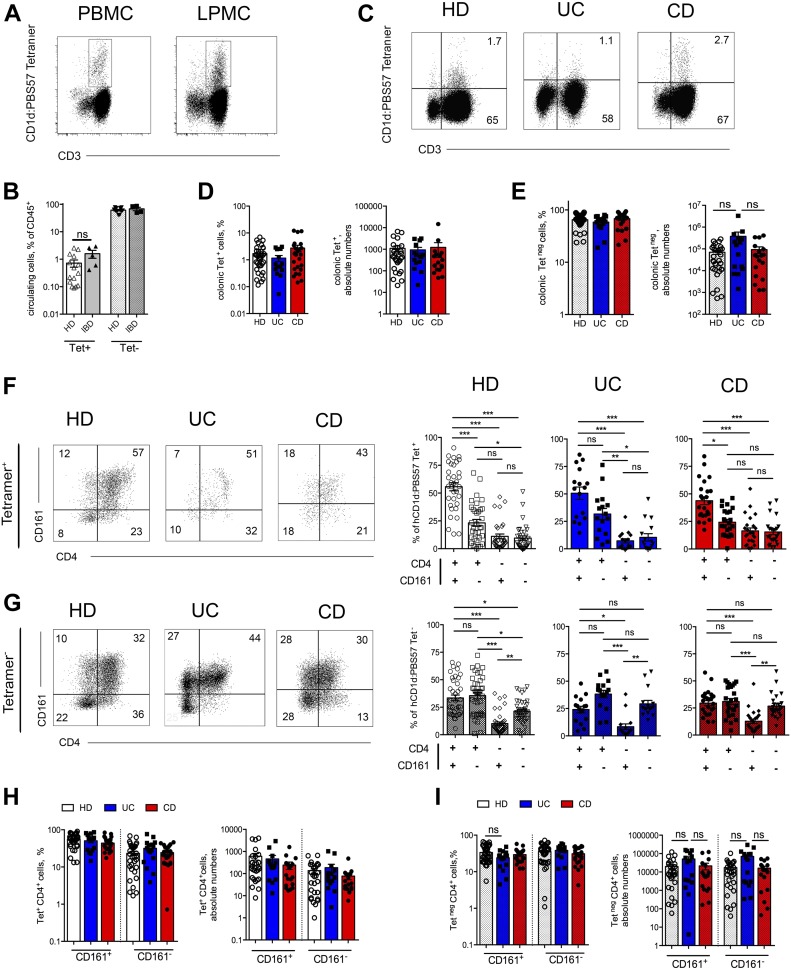
LP iNKT cells express CD4 and CD161 and secrete pro-inflammatory cytokines. **(A, B)** Representative dot plots (A) and summary of frequencies (B) of circulating iNKT (hCD1d:PBS57 tetramer^+^, plain bars) and of conventional T cells (hCD1d:PBS57 tetramer^−^, dotted bars) among total CD3^+^ lineage cells in the peripheral blood (PB) of HDs (n = 15) or IBD patients (n = 5). **(C–E)** Representative dot plots (C), summary of frequencies (D and E, left panels), and absolute numbers (D and E, right panels) of colonic iNKT (D) and of conventional T cells (E) in the LP of uniflamed donors (HDs, white bars, n = 27), UC patients (blue bars, n = 16), and CD patients (red bars, n = 24). **(F, G)** Representative dot plots (right panels) and cumulative frequencies of CD4/CD161–expressing cells among iNKT (tetramer^+^, F) and conventional T cells (tetramer^−^, G) isolated from the LP of HDs (left panels), UC patients (middle panels), and CD patients (right panels). **(H, I)** Comparison between CD161+ and CD161− expressing cells among CD4^+^ iNKT (H, tetramer^+^) and conventional T cells (I, tetramer^−^) isolated from the LP of HDs (white bars, n = 27), UC patients (blue bars, n = 16), and CD patients (red bars, n = 24). Left panels, frequencies; right panels, absolute numbers. Statistical significance was calculated using the Kruskal–Wallis nonparametric test for multiple comparisons. *P* < 0.05 (*), *P* < 0.01, (**), *P* < 0.001 (***) were regarded as statistically significant. Error bars: mean ± SEM.

**Figure S1.**
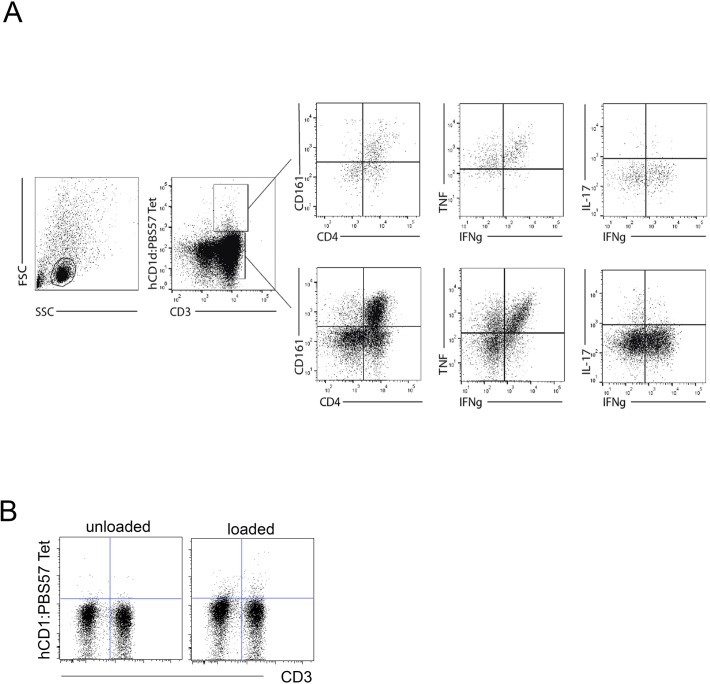
Gating strategy to identify iNKT cells amongst LPMCs and PMBCs. **(A)** From left, forward and side scatter to identify lymphocytes, CD3/hCD1d:PBS57 tetramer expression. Among CD3+Tet+ or CD3+Tet-cells, expression of CD4 and CD161. On the different subsets, intracellular cytokines (IL-17A, IFNγ, TNF, and IL-13). **(B)** Dot plots indicate iNKT cells staining with unloaded (left panels) or PBS57-loaded hCD1d tetramer.

Different T lymphocyte subsets are recruited in the intestinal LP during the processes associated with IBD-related inflammation (Kaser et al, 2010). To initially address the contribution of type I iNKT cells in human IBD, the frequency of circulating (Fig 1A) and intestinal iNKT cells (Fig 1B) was analyzed ex vivo from individuals affected by both UC (n = 16) and CD (n = 24) or noninflammatory IBD-unrelated intestinal pathologies in healthy donors (HDs) (n = 27) (Table 1). LP mononuclear cells (LPMCs) were isolated from surgical specimens of the different subgroups, and iNKT cells were identified by hCD1d:PBS57 tetramer recognition (Fig S1A), whose specificity was confirmed by unloaded CD1d tetramer staining (Fig S1B).

**Table 1.** Patients description.

Clinical parameter	Healthy controls, n = 27	UC, n = 16	CD, n = 24
Male/female, n	14/13	9/7	12/12
Age at enrolment, mean ± SD, yr	69.5 (±12.31)	40.75 (±13.3)	39.61 (±7.37)
Disease duration, mean ± SD, yr	—	8.8 (±4.8)	10.5 (±6.21)
Smoking status, yes/no/ex	—	2/13/1	3/19/2
CD, n			
L1 ileal	—	—	10
L2 colonic	—	—	3
L3 ileocolonic	—	—	11
L4 upper	—	—	0
B1 (not strict/not penetrating)	—	—	6
B2 (stricturing)	—	—	13
B3 (penetrating)	—	—	5
UC, n			
E1 proctitis	—	1	—
E2 left-sided	—	11	—
E3 pancolitis	—	4	—
Concomitant therapy at enrolment			
No therapy	—	7	15
Antibiotics, n	—	0	1
Mesalamine, n	—	6	3
Thiopurines, n	—	—	5
Corticosteroids, n	—	3	2
Anti-TNF	—	—	2

As previously observed for other tissues (Chan et al, 2013), LP iNKT cells displayed high variability in frequency, spanning from 0.1 to 10% of the total CD3^+^ T lymphocytes, and in absolute numbers (Fig 1C and D). iNKT cells were slightly more abundant in the intestinal LP than in the peripheral blood (Fig 1A–D), in accordance with the notion that most iNKT cells do not circulate but localize preferentially within tissues (Kim et al, 2002). iNKT cells and tetramer-negative CD3^+^ T cell frequencies and absolute numbers were not substantially different between IBD patients and non-IBD donors (Fig 1C–E).

**Figure S2.**
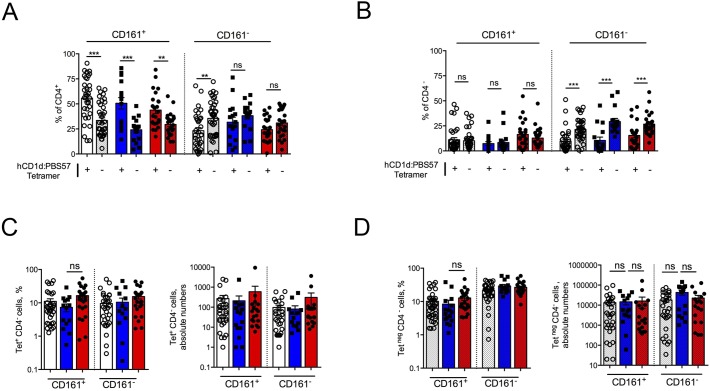
CD161 expression comparison between intestinal tetramer^+^ and tetramer^−^ cells. **(A, B)** CD161 expression among CD4^+^ (A) and CD4^−^ (B) tetramer^+^ (+, plain bars) or tetramer-negative (−, dotted bars) cells isolated from HDs (HD, white bars), UC patients (blue bars), and CD patients (red bars). **(C, D)** Comparison of CD161 expression and absolute numbers among CD4^−^ tetramer^+^ (C) or tetramer-negative (D) cells isolated from HDs (HD, white bars), UC patients (blue bars), and CD patients (red bars).

Co-expression by LP T lymphocytes of CD4 and CD161, a tissue-homing integrin highly expressed by gut-tropic T cells, has been associated with pathogenic functions in IBD patients (Fuss et al, 2004; Annunziato et al, 2008). Almost 80% of intestinal iNKT cells were CD4^+^ (Figs 1F and S2), and more than half co-expressed CD161 (Fig 1F and H). Likewise, tetramer-negative CD3^+^ T cells were mainly CD4^+^, and CD161 expression analysis confirmed previously published data in CD (Annunziato et al, 2008) and UC patients (Fuss et al, 2004, Fuss et al, 2014) (Figs 1G and I, and S2). Interestingly, CD161^+^ and CD161^−^ CD4^+^ iNKT cell populations were equally represented in UC patients (Figs 1H and S2), similarly to what observed for CD4^+^ tetramer-negative cells (Figs 1I and S2), whereas in HDs and CD patients, iNKT cells were mostly CD161^+^.

**Figure 2.**
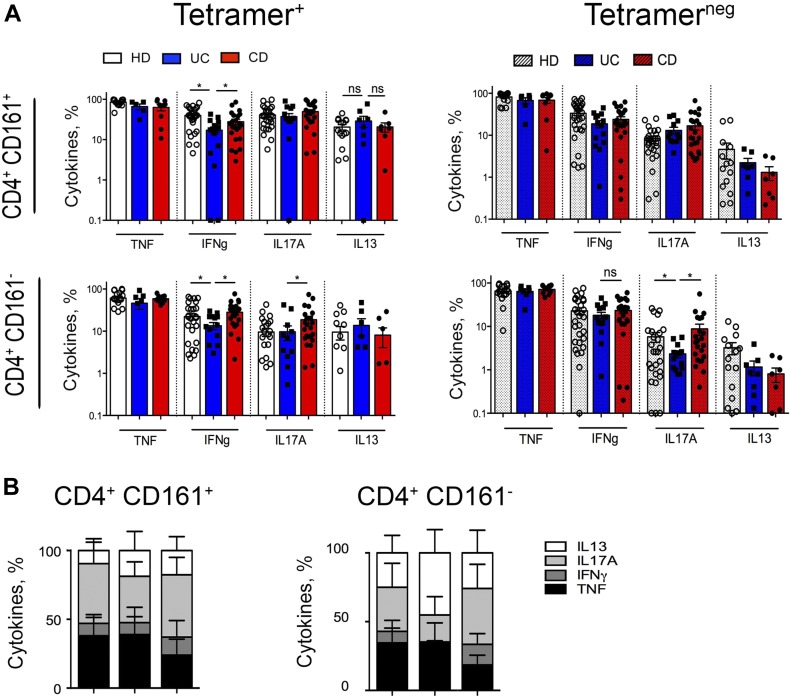
Mucosal iNKT secrete pro-inflammatory cytokines. **(A)** Comparison between pro-inflammatory cytokines (TNF, IFNγ, IL17A, and IL13) produced upon brief polyclonal stimulation by CD161+ and CD161− expressing cells among CD4^+^ iNKT (left panels) and conventional T cells (right panels) isolated from the LP of HDs (white bars, n = 27), UC patients (blue bars, n = 16), and CD patients (red bars, n = 24). Statistical significance was calculated using the Kruskal–Wallis nonparametric test for multiple comparisons. *P* < 0.05 (*), *P* < 0.01 (**) were regarded as statistically significant. Error bars: mean ± SEM. **(B)** Frequency of pro-inflammatory cytokines (TNF, IFNγ, IL17A, and IL13) produced by unstimulated CD4^+^ CD161^+^ (left graphs) or CD4^+^CD161^−^ (right graphs) intestinal ex vivo–isolated iNKT cells (tetramer^+^) from HDs (n = 27), UC patients (n = 16), and CD patients (n = 24). Error bars: mean ± SEM.

**Figure S3.**
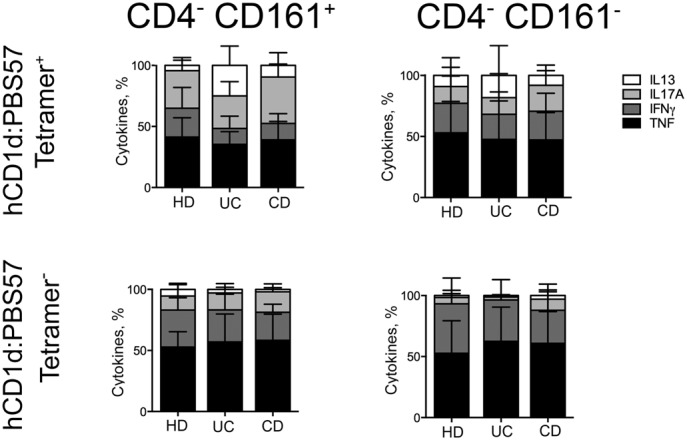
Pro-inflammatory cytokine secretion by CD4^−^ Tetramer^+^ and Tetramer^−^ T cells. Frequency of pro-inflammatory cytokines (TNF, IFNg, IL17A, and IL13) produced upon brief polyclonal stimulation by CD4^−^CD161+ (left graphs) or CD4^−^CD161^−^ (right graphs) intestinal ex vivo–isolated iNKT cells (tetramer+, upper panels) or conventional T cells (tetramer−, lower panels) from HDs (n = 27), UC patients (n = 16), and CD patients (n = 24).

In previous studies, the expression of the CD161 marker in conventional T helper cells from CD patients identifies IL-17–secreting T cell subset (Th17 cells) (Cosmi et al, 2008). Here, we found that both iNKT and conventional CD4^+^ T cells CD161^+^ cells secreted IL-17 (Figs 2 and S3 and Tables S1 and S2), and also TNF and IFNγ, especially in CD patients (Fig 2A). CD161^+^ iNKT cells secrete moderate levels of IL-13 when polyclonally restimulated ex vivo (Figs 2A and S3), especially those derived from UC patients, but similar findings were observed from CD161-iNKT isolated from UC and CD patients (Fig 2A). Interestingly, ex vivo–isolated iNKT cells constitutively secreted relevant amounts of cytokines, in particular IL-17, IL-13, and TNF (Fig 2B), even in the absence of in vitro restimulation, as if they were already pre-stimulated in vivo.

Table S1 Statistical summary of iNKT cell cytokine production.

Table S2 Statistical summary of conventional T cell cytokine production.

**Figure S4.**
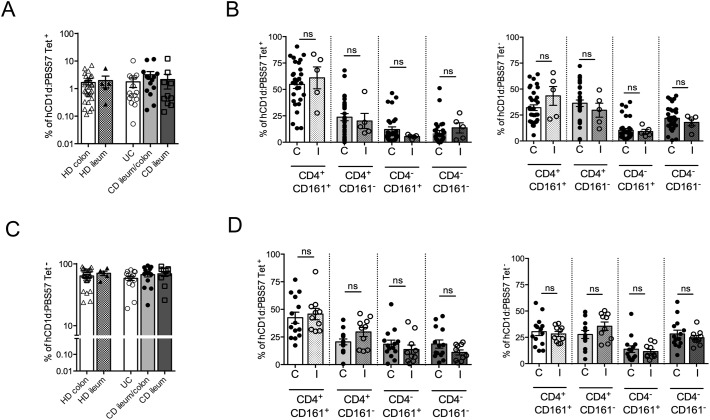
Analysis of frequency and phenotype according to colonic or ileal localization. **(A, C)** Frequency of tetramer+ (A) or tetramer− (C) CD3^+^ LPMC subdividing patients for colonic or ileal disease localization. **(B, D)** Frequency of CD161/CD4 expressing tetramer+ (left panel) or tetramer− (right panels) CD3^+^ LPMC subdividing HDs (B) and CD (D) patients for colonic (C) or ileal (I) disease localization. HD colon, n = 22; HD ileum, n = 5; CD colon, n = 13; CD ileum/colon, n = 11.

To note, colonic or ileal CD localization did not substantially influence neither the frequency nor the phenotype of LP iNKT cells (Fig S4).

Taken together, these data indicate that phenotypically distinct subsets of iNKT cells are present in the LP of HDs and IBD patients and that pro-inflammatory cytokines are secreted by these cells in the intestinal mucosa.

### Intestinal iNKT cell lines and clone generation

**Figure 3.**
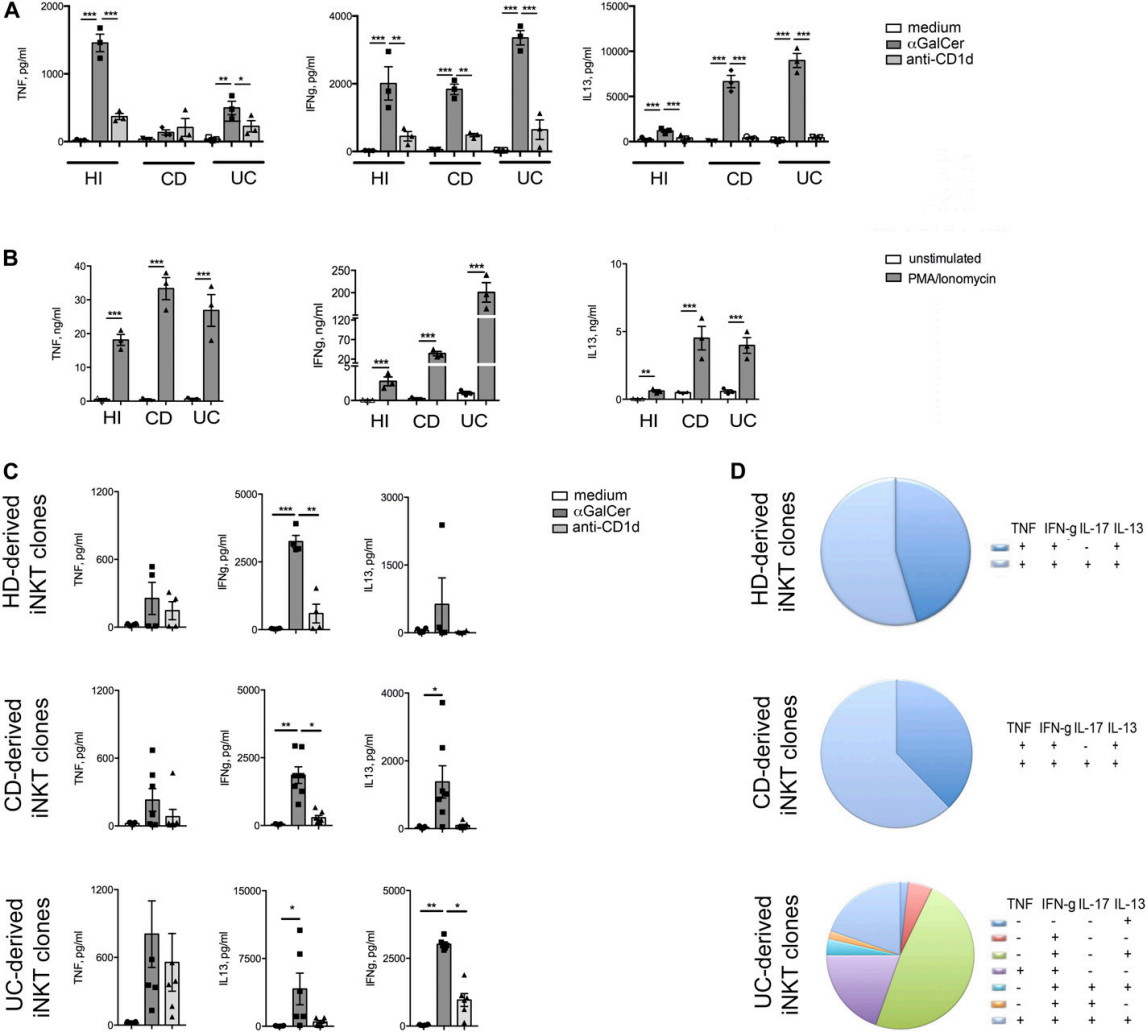
Human intestinal iNKT cell line and clones generation. **(A)** TNF, IFNγ, and IL-13 secretion upon co-culture of iNKT cell lines with moDC in the absence (white bars) or presence (dark grey bars) of aGalcer and of concomitant CD1d blocking (light grey bars). **(B)** Cytokine measurement in the supernatants of iNKT cell lines polyclonally stimulated (PMA/ionomycin, 3 h in the absence of Brefeldin A). **(C)** TNF, IFNγ, and IL-13 production by representative iNKT cell clones (HD, n = 4; CD, n = 7; UC, n = 6) upon αGalCer stimulation in the presence (light grey bars) or absence (dark grey bars) of anti hCD1d-blocking antibodies. White bars, medium only. **(D)** Cumulative FACS analysis of the cytokine profile of 10 HD (out of 12), 50 UC (out of 196), and 50 CD (out of 210) intestinal-derived iNKT cell clones upon 3 h PMA/ionomycin stimulation (in the presence of Brefeldin A). Statistical significance was calculated using the Kruskal–Wallis nonparametric test for multiple comparisons. *P* < 0.05 (*), *P* < 0.01 (**), *P* < 0.001 (***) were regarded as statistically significant. Error bars: mean ± SEM.

**Figure S5.**
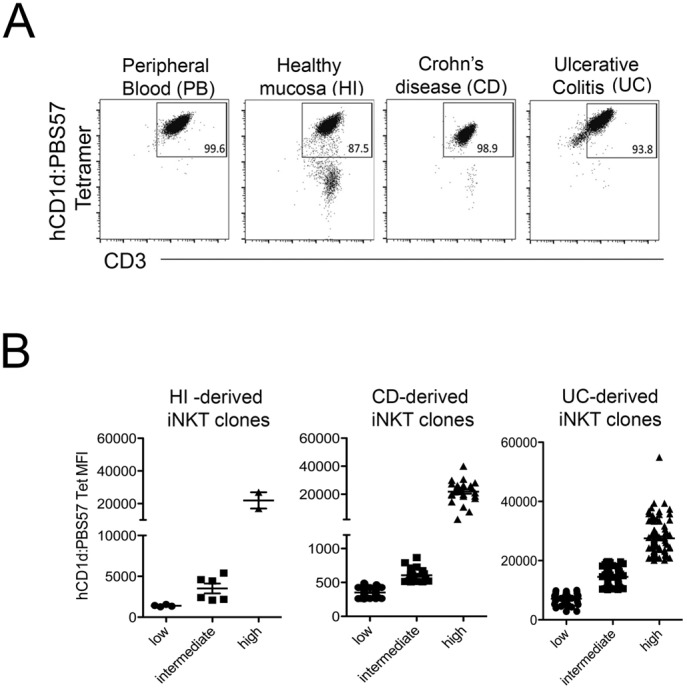
**(A)** Representative dot plots of human iNKT cell lines derived from healthy mucosa (HD), UC, and CD surgical specimens. **(B)** Distribution of HD (left panel, n = 12), CD (middle panel, n = 192), and UC (left panel n = 300) clones according to binding to hCD1d:PBS57 tetramer.

**Figure S6.**
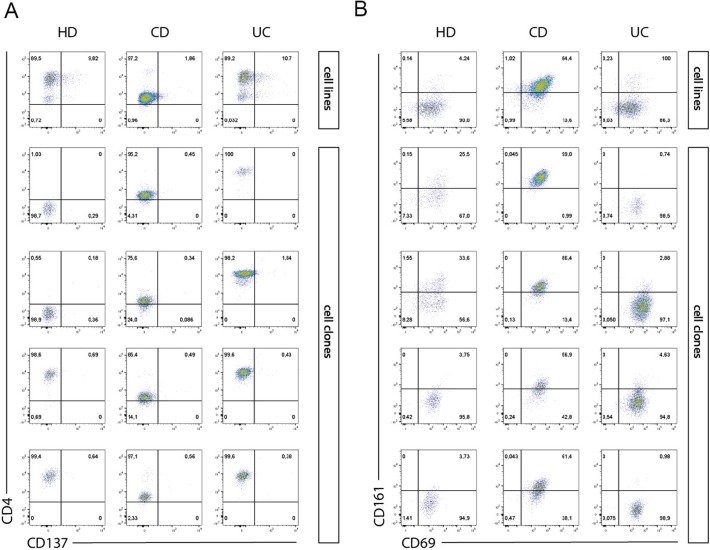
Phenotypical characterization of representative human iNKT cell lines and clones. **(A, B)** Representative dot plots of CD4, CD137 (A), CD161, and CD69 (B) surface expression in PB-, CD-, HI-, and UC-derived iNKT cell lines (upper panels) and four different clones (lower panels; left: HD-derived, middle: CD-derived, right: UC-derived).

**Figure S7.**
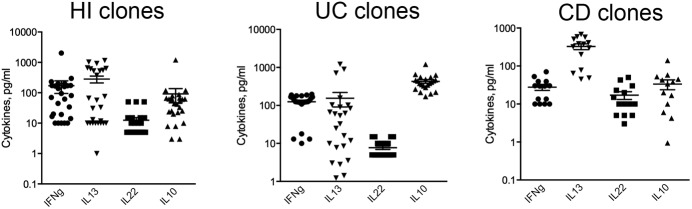
Basal cytokine production by iNKT cell clones. IFNg, IL13, IL22, and IL10 basal production of unstimulated iNKT cell HI- (left panel), UC- (middle panel), and CD- (right panel) derived iNKT cell clones.

To study the functional behavior of human intestinal iNKT cells, stable cell lines and clones were generated from total LPMCs isolated from inflamed intestinal tissue of IBD patients (UC and CD) or from not inflamed intestinal surgical specimens (HDs) (Figs 3 and S5A and B). Their phenotype (Fig S6) and the basal cytokine profile (Fig S7) reflected the expression of CD4, CD161, and cytokine secretion of ex vivo–isolated iNKT cells, were fully functional, and responded both to antigen-specific (Fig 3A) and polyclonal (Fig 3B) stimulation with pro-inflammatory cytokine secretion.

More than 400 intestinal iNKT cell-independent clones were also generated from IBD (UC and CD) patients and HDs. These clones were characterized by different expression levels of TCR expression evaluated as PBS57:CD1d tetramer staining intensity (Fig S5B). Similarly to what we observed with iNKT cell lines, antigen-specific (Fig 3C) and polyclonal stimulation (Fig 3D) induced a potent secretion of pro-inflammatory cytokines also on iNKT cell clones (Fig 3C). Interestingly, polyclonally stimulated UC-derived clones showed a more heterogeneous cytokine profile when compared with HDs or CD-derived ones (Fig 3D). To note, IL17 production by iNKT cells is hardly detectable by ELISA.

**Figure 4.**
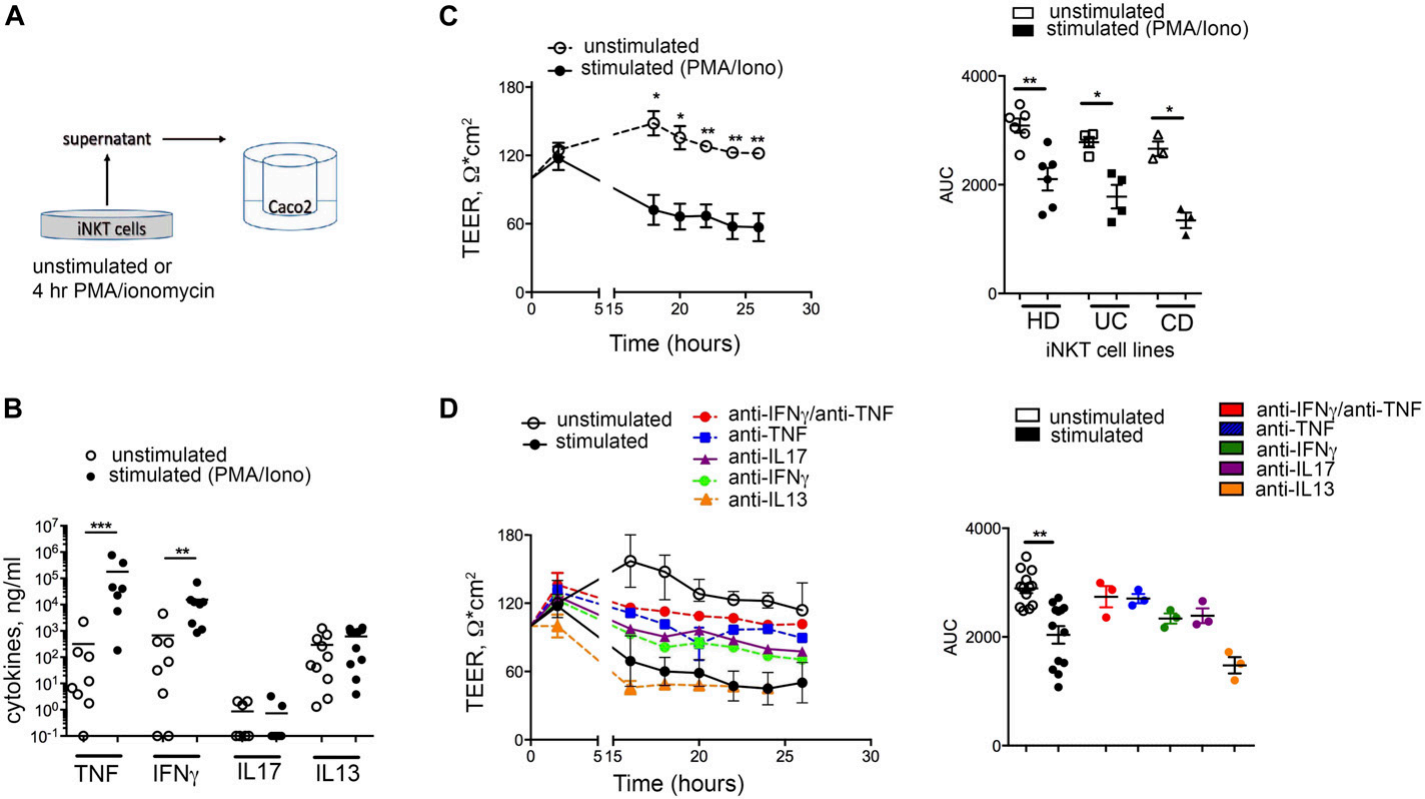
Human iNKT cells are pathogenic against epithelial cells. **(A)** Schematic representation of the experiment. **(B)** Multiplex analysis of TNF, IFNγ, IL-17A, and IL-13 concentrations in the supernatants of polyclonally stimulated iNKT cells. **(C)** TEER measured upon co-culture of Caco-2 cells with supernatants of unstimulated (open circles) or polyclonally stimulated (closed circles) iNKT cells. Left panel, representative plot; right panel, area under the curve (AUC) of n = 5 independent experiments with iNKT lines. **(D)** TEER measured upon co-culture of Caco-2 cells with supernatants of unstimulated (open circles) or polyclonally stimulated iNKT cell lines in the absence (closed circles) or presence of anti IFNγ/TNF (red circles), anti TNF (blue circles), anti IL17 (purple circles), anti IFNγ (green circles), and anti IL13 (orange circles) inhibitors. Left panel, representative plot; right panel, AUC of n = 3 independent experiments. Statistical significance was calculated using the Kruskal–Wallis nonparametric test for multiple comparisons. *P* < 0.05 (*), *P* < 0.01 (**), *P* < 0.001 (***) were regarded as statistically significant. Error bars: mean ± SEM.

Finally, to test if intestinal iNKT cells might acquire pathogenic functions against the intestinal epithelium upon activation, iNKT cells were polyclonally stimulated and their supernatants, containing pro-inflammatory cytokines (Fig 4B), were applied in vitro to polarized epithelial Caco-2 monolayers (Fig 4A). Activated iNKT cells, independently from their origin, manifested a pathogenic potential affecting epithelial cell monolayer integrity, as demonstrated by decrease of trans-epithelial electrical resistance (TEER) (Fig 4C). Similarly to conventional Th17 cells (Nizzoli et al, 2018), this effect could be inhibited upon neutralization of several T cell cytokines, with the notable exclusion of IL-13 (Fig 4D).

Collectively, these data indicate that the functional phenotype of intestinal iNKT cell lines and clones reflects that of ex vivo–isolated intestinal iNKT cells. Hence, these lines and clones can be used as an innovative tool to study intestinal iNKT cells in vitro and assess their potential contribution to gut inflammation.

### iNKT cells respond to mucosa-associated microbiota

A current hypothesis holds that aberrant activation of pathogenic T lymphocytes in IBD patients depends on gut microbiota recognition, and it is known that gut microbes are potent stimulators of iNKT cell responses (Tupin et al, 2007; Kamada & Nunez, 2013; Strober, 2013). Hence, we asked whether the gut microbiota directly activates human intestinal iNKT cells leading to the pro-inflammatory phenotype of iNKT cells in IBD patients.

**Figure 5.**
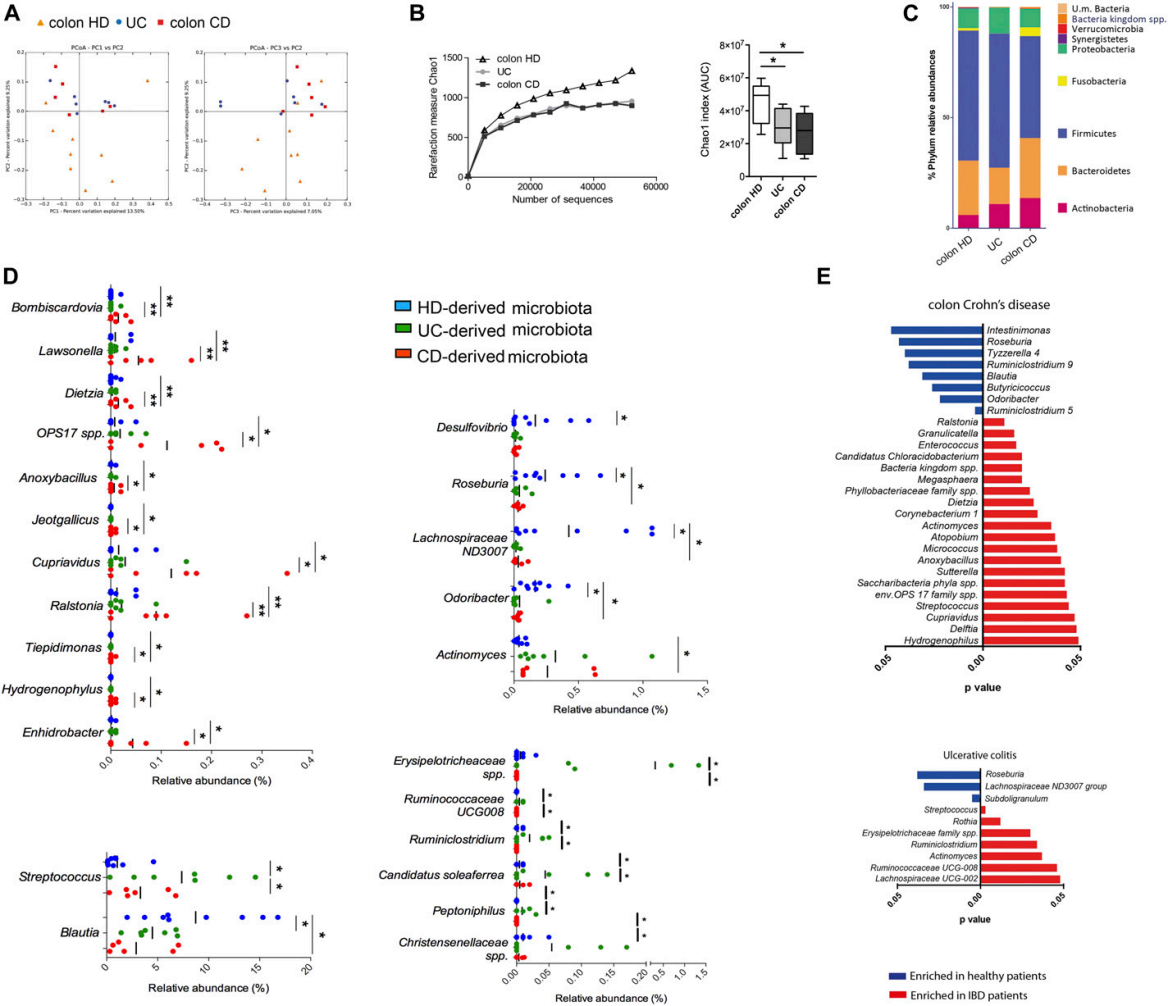
Mucosa-associated microbiota analysis in IBD patients and HDs. **(A)** Microbiome clustering based on unweighted principal coordinate analysis (PCoA) UniFrac metrics of mucosa-associated microbiota derived from HDs (orange triangles), UC patients (blue dots), and colon CD (red squares) patients. **(B)** Rarefaction curves and cumulative AUC showing microbial richness based on the Chao1 index. **(C)** Bar plots of the taxonomic composition showing relative abundances >1% of bacterial phyla. **(D)** Relative abundance of OTUs differentially present in the mucosa-associated microbiota derived from HDs (blue dots), UC patients (green dots), and CD patients (red dots) compared with the other groups. **(E)** Comparison of the relative abundances of different taxa between colon CD patients (left panels) or UC patients (right panels) and HDs. Blue bars, taxa enriched in HD; red bars, taxa enriched in IBD patients. Statistical significant differences were assessed through the Mann–Whitney test for comparisons between two groups or with the Kruskal Wallis test with least significant difference post hoc test for more than two groups. *P < 0.05, **P < 0.01. Error bars: mean ± SEM.

**Figure S8.**
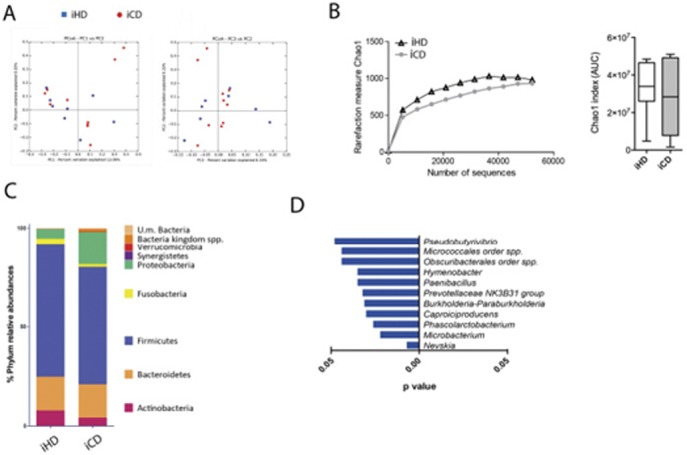
Mucosa-associated microbiota analysis of ileal CD and HD donors. **(A)** Microbiome clustering based on unweighted principal coordinate analysis (PCoA) UniFrac metrics of mucosa-associated microbiota derived from ileal HDs (blue dots) and ileal CD (red dots) patients. **(B)** Rarefaction curves and cumulative AUC showing microbial richness based on the Chao1 index. **(C)** Bar plots of the taxonomic composition showing relative abundances >1% of bacterial phyla. **(D)** Comparison of relative abundances of different taxa between ileal CD patients (left panels) and HDs. Blue bars, taxa enriched in HDs. Statistical significant difference was assessed through one-way ANOVA with least significant difference post hoc test.

To this aim, the gut mucosa-associated microbiota was collected from surgical specimens of IBD patients and HDs, and the bacterial composition was evaluated by 16S rRNA sequencing (Fig 5). Unweighted UniFrac-based comparisons of the samples isolated from the colon of 9 HDs, 7 UC patients, and 6 colonic CD patients (Fig 5A), as well as from the ileum of 8 HDs and 10 ileal CD patients (Fig S8) were performed. Principal coordinates analysis (PCoA) differentiated healthy microbiota samples from IBD patients, but no differences between UC and CD samples were detected (Fig 5A). Also, mucosa-associated microbiota derived from IBD patients showed a lower α-diversity when compared with HD microbiota (Pascal et al, 2017; Gevers et al, 2014) (Fig 5B). The taxonomic composition of the mucosa-associated microbiome of IBD patients showed an increase of *Proteobacteria* and *Fusobacteria* and a decrease in *Firmicutes* compared with HDs. As previously reported (Kim & Chung, 2013; Pascal et al, 2017), these alterations were more evident in microbiota samples from CD as compared with UC patients (Figs 5C and S8). To note, around 15% of the microbial ecology at genus level was significantly changed in the samples analyzed (Fig 5D). Specific variations between colonic IBD and HD-derived samples included the increase of *Actinomyces* and *Streptococcus* and the decrease of *Roseburia*, *Blautia*, *Desulfovibrio*, *Odoribacter*, and *Lachnospinaceae ND3007* (Fig 5D and E); specific CD-associated changes included increase of *Lawsonella*, *Dietzia*, *Ralstonia*, and *Enhidrobacter*, whereas UC-associated changes included increase of *Erysipelotricheaceae*, *Ruminococcaceae*, *Ruminoclostridium*, and *Christanesellacee* (Fig 5D and E).

**Figure 6.**
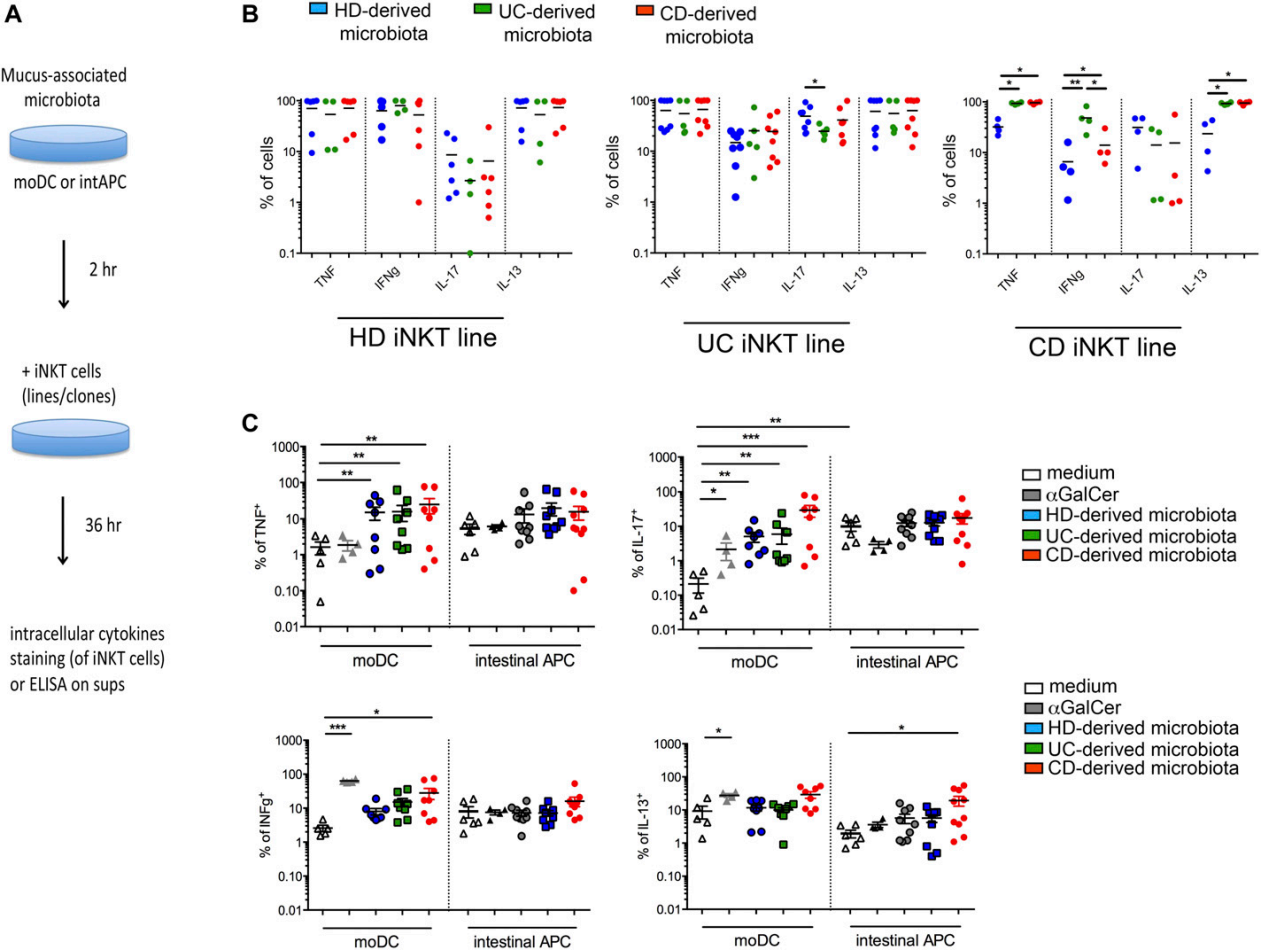
iNKT cells react to mucosa-associated microbiota. **(A)** Schematic representation of the experiment. **(B)** Cytokine profile of iNKT cell lines (HD, UC, and CD) co-cultured 36 h with monocyte-derived dendritic cells exposed to 100 ng of mucosa-associated microbiota isolated from 8 HDs (blue dots), 7 UC patients (green dots), and 8 CD patients (red dots). **(C)** Frequency of TNF, IFNγ, IL17, and IL13 positive iNKT cells co-cultured 36 h with moDC or ex vivo–sorted CD1d^+^MHCII^+^CD3^−^ intestinal APCs in the absence (white dots) or presence of aGalCer (grey dots) or to 100 ng of mucosa-associated microbiota isolated from at least 6 HDs (blue dots), 4 UC patients (green dots), and 6 CD patients (red dots) in three independent experiments. Statistical significance was calculated using the Kruskal–Wallis nonparametric test for multiple comparisons. *P* < 0.05 (*), *P* < 0.01 (**), *P* < 0.001 (***) were regarded as statistically significant. Error bars: mean ± SEM.

**Figure S9.**
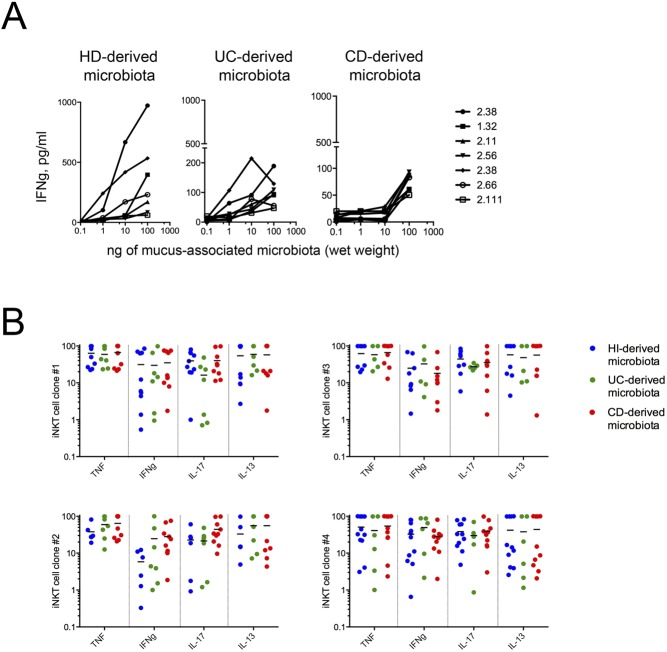
iNKT cells functionally respond to mucosa-associated microbiota. **(A)** IFNg production by different iNKT cells clones co-cultured with moDC exposed to mucosa-associated microbiota derived from one HD (left panel), one UC (middle panel) patient, and one CD (right panel) patient. **(B)** Cumulative representation of TNF, IFNG, IL17, and IL13 produced by four different clones co-cultured with moDC exposed to eight different HD (blue dots), seven different UC (green dots), and eight different CD (red dots) mucosa-associated microbiota samples. Statistical significant difference was assessed through one-way ANOVA with least significant difference post hoc test *P < 0.05.

To test whether the pro-inflammatory phenotype of ex vivo–analyzed iNKT cells was linked to the distinguished gut microbiota profile of IBD patients, we exposed iNKT cells (lines and clones) in vitro to the mucosa-associated microbiota samples from IBD and HDs that we had characterized by metagenomic analysis (Fig 6A). All the iNKT cell lines (Fig 6B) and the clones (Fig S9B and data not shown) cultured with monocyte-derived dendritic cells in the presence of gut microbiota, regardless its origin, were activated and secreted high amounts of pro-inflammatory cytokines such as TNF, IFNγ, and IL-13. Nonetheless, qualitatively and quantitatively different responses were observed when iNKT cell lines were exposed to the mucosal microbiota isolated from IBD patients compared with HDs (Fig S9A, representation of experiments with one microbiota donor per group; Fig 6B quantification of pulled experiments with n = 6/8 independent donors per group). These results are in line with the overall complexity of the microbiota ecology and with the possible presence of different TCR specificities among iNKT cell lines (Cameron et al, 2015). Interestingly, also HD-derived microbiota stimulated iNKT cells and induced the secretion of pro-inflammatory cytokines. However, exposure of CD-derived iNKT cell lines to IBD-derived microbiota triggered a strong production of pro-inflammatory cytokines. Conversely, exposure of iNKT cell lines derived from the intestinal tissue of HDs to the microbiota samples (from IBD or HD) did not result in a marked pro-inflammatory cytokine skewing. In addition, IL-17 secretion by UC iNKT cell lines was decreased upon exposure to UC-derived microbiota (Fig 6B).

**Figure S10.**
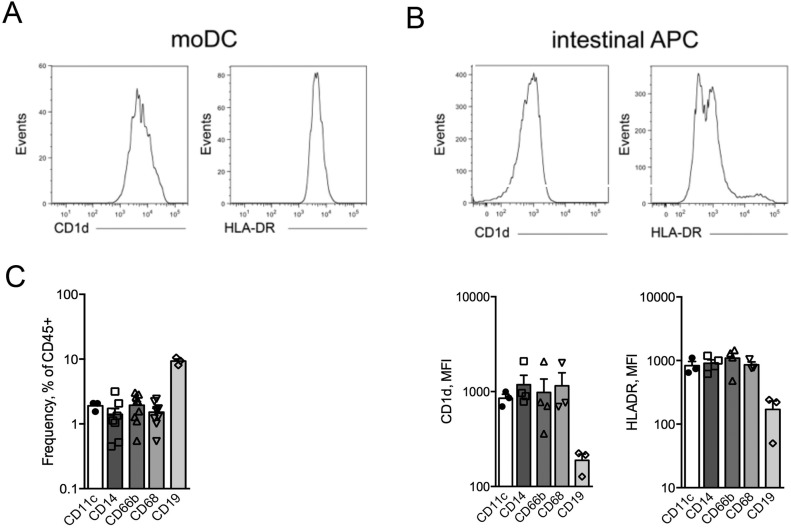
CD1d and HLADR levels on moDC and intestinal APCs. **(A, B)** CD1d (left panels) and HLA-DR (right panels) in moDC (A) and sorted intestinal APCs (B upper panels, histograms; B lower panels, cumulative MFI of four independent experiments). **(C)** Plot representing the frequency of dendritic cells, monocytes, neutrophils, macrophages, and B cells in sorted LPMCs from 4 to 6 independent experiments.

Next, we tested whether the microbiota-induced pro-inflammatory effect on iNKT cells associated with APC modulation. In vitro–differentiated monocyte-derived dendritic cells (moDC) or ex vivo–sorted CD1d^+^ HLADR^+^ intestinal APCs (composed by macrophages, dendritic cells, monocytes, as well as few granulocytes and B cells; Fig S10) were exposed to mucosa-associated microbiota samples, and then their capacity to induce different cytokine profiles in iNKT cells was evaluated. The exposure of CD1d^+^ sorted intestinal APCs, as compared with moDC, to gut microbiota induced a preferential Th1/Th17 response (Caprioli et al, 2013) and a relevant IL13 production by iNKT cells, whereas only those challenged with CD-derived microbiota induced a sustained IFNγ secretion (Fig 6C). To note, unstimulated intestinal APCs were sufficient to induce an IL17 response by iNKT cells.

In conclusion, iNKT cells become functionally activated upon exposure to human mucosa-associated microbiota and display a highly skewed pro-inflammatory phenotype.

### iNKT cells recognize intestinal pathobionts by innate and adaptive mechanisms and become pathogenic against intestinal epithelial cells

**Figure 7.**
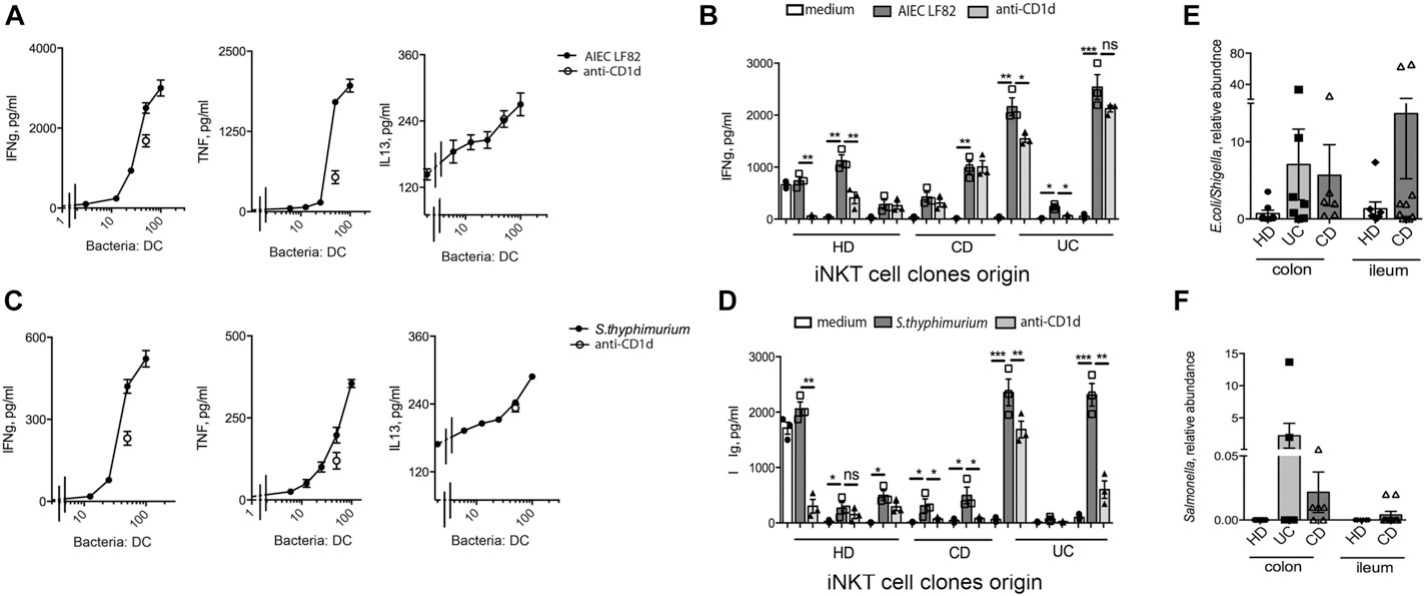
Human iNKT cells respond to stimulation with pathogenic intestinal bacterial strains. **(A, C)** IFNγ (left panels), TNF (middle panels), and IL-13 (right panels) in the supernatants of iNKT cells co-cultured 36 h with monocyte-derived dendritic cells exposed to increased doses of the *adherent-invasive E. coli* LF82 (AIEC, A) and of *Salmonella typhimurium* (C). Light grey bars, stimulation in the presence of anti-CD1d blocking antibody. **(B, D–F)** Cumulative representation of IFNg production upon exposure to AIEC (B) or *Salmonella typhimurium* (D) in at least three independent experiments with two different HD clones, three different CD clones, and three different UC clones (E, F). *Escherichia*/*Shigella* (E) and *Salmonella* (F) genus in colonic HDs (n = 9), UC (n = 7), colonic CD (n = 6), ileal HD (n = 7), and ileal CD (n = 7). Statistical significance was calculated using the Kruskal–Wallis nonparametric test for multiple comparisons. *P* < 0.05 (*), *P* < 0.01 (**), *P* < 0.001 (***) were regarded as statistically significant. Error bars: mean ± SEM.

**Figure S11.**
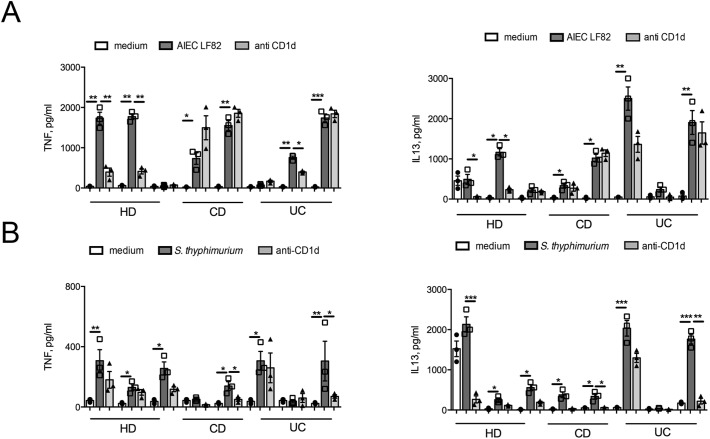
iNKT cells respond to stimulation with intestinal pathogenic bacteria. **(A, B)** Cumulative representation of TNF (left panels) and IL13 (right panels) in the supernatants of iNKT cell clones co-cultured 36 h with monocyte-derived dendritic cells infected with *adherent-invasive E. coli* LF82 (AIEC, A) and of *Salmonella typhimurium* (B) in at least three independent experiments with two different HD clones, three different CD clones, and three different UC clones. Statistical significance was calculated using the Kruskal–Wallis nonparametric test for multiple comparisons. *P* < 0.05 (*), *P* < 0.01 (**), *P* < 0.001 (***) were regarded as statistically significant.

Functional activation of iNKT cells is mediated by CD1d-dependent and/or independent mechanisms (Bendelac et al, 2007). To explore whether microbiota-induced iNKT cell activation requires CD1d presentation, we exposed iNKT cells in vitro to two well-characterized purified intestinal pathobionts, known to be present in IBD patients and to mediate inflammatory responses (Schultz et al, 2017; Palmela et al, 2018), that is, adherent invasive *Escherichia coli* (AIEC) strain LF82 (Figs 7A and B and S11A) and *Salmonella typhimurium* (Figs 7C and D and S11B). Both strains induced a potent dose-dependent pro-inflammatory activation of iNKT cells, resulting from a combination of both antigenic and non-antigenic (innate) stimulation, as demonstrated by its only partial inhibition after CD1d blockade (Figs 7A–D and S11).

Noteworthy, in our cohort of patients, the genus *Salmonella* was detected almost exclusively in mucus-associated IBD samples (Fig 7F), whereas the *Escherichia/Shighella* genus was detected also at lower levels in HD samples (Fig 7E). However, iNKT cell lines and clones of different origin were similarly activated in vitro with all the bacteria tested, including the laboratory *E. Coli* strain HB101 (data not shown).

**Figure 8.**
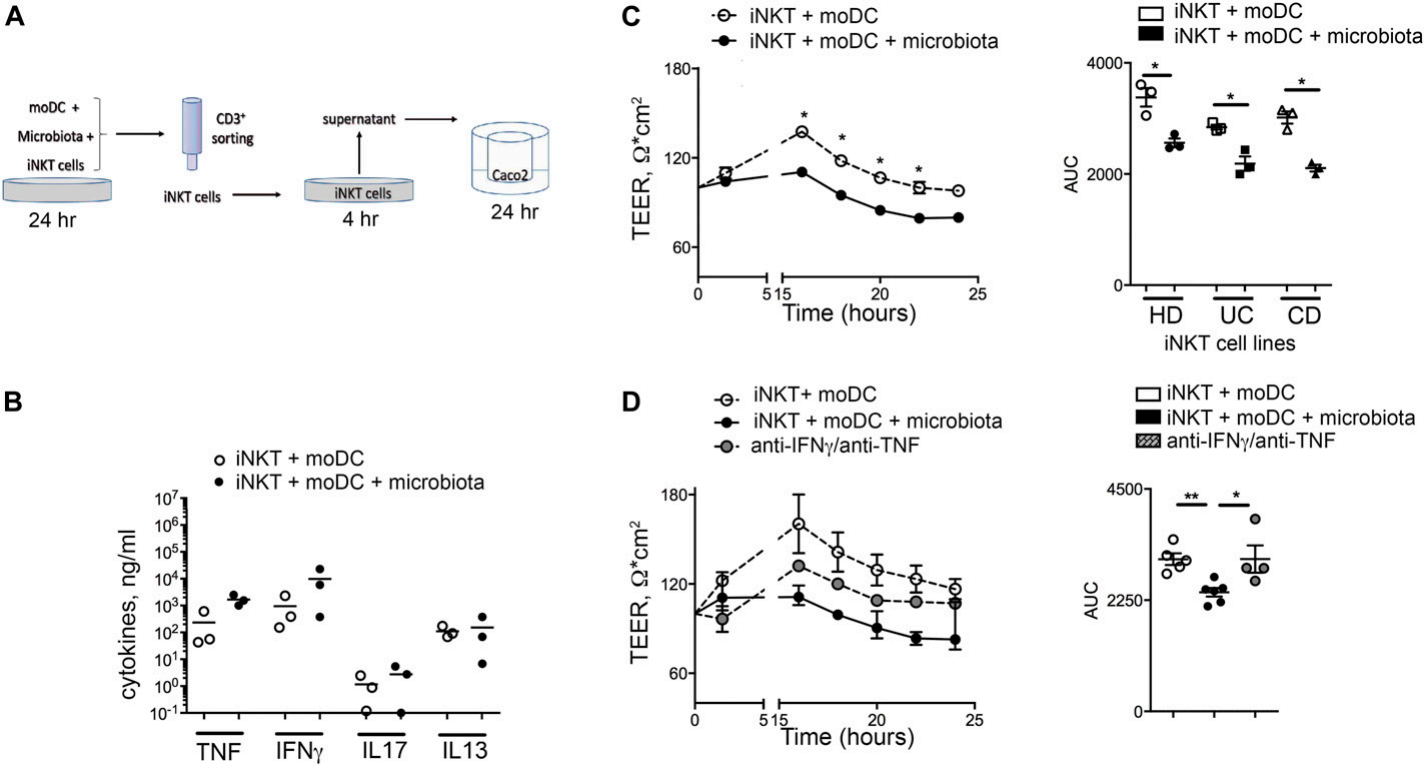
Microbiota-stimulated human iNKT cells are pathogenic against epithelial cells. **(A)** Schematic representation of the experiment. **(B)** Multiplex analysis of TNF, IFNγ, IL-17A, and IL-13 concentrations in the supernatants of iNKT cells co-cultured with moDC alone (open circles) or with moDC stimulated with intestinal microbiota (closed circles). **(C)** TEER measured upon co-culture of Caco-2 cells with supernatants of iNKT cells co-cultured with moDC alone (open circles) or with moDC stimulated with intestinal microbiota (closed circles). Left panel, representative plot; right panel, area under the curve (AUC) of n = 4 independent experiments with iNKT cell lines. **(D)** TEER measured upon co-culture of Caco-2 cells with supernatants of iNKT cells with moDC alone (open circles) or with moDC stimulated with intestinal microbiota (closed circles) in the absence (closed circles) or presence of anti IFNγ/TNF (grey circles) inhibitors. Left panel, representative plot; right panel, AUC of n = 3 independent experiments. Statistical significance was calculated using the Kruskal–Wallis nonparametric test for multiple comparisons. *P* < 0.05 (*), *P* < 0.01 (**), *P* < 0.001 (***) were regarded as statistically significant. Error bars: mean ± SEM.

Finally, to test if exposure to the mucosa-associated microbiota might induce specific pathogenic functions against the intestinal epithelium, iNKT cells were exposed to the mucosa-associated IBD-derived microbiota (Fig 8A). Consistent to our previous findings, microbiota-activated iNKT cells secreted pro-inflammatory cytokines (Fig 8B).

Microbiota-activated iNKT cells, regardless of their origin, manifested pathogenic activities affecting epithelial cell monolayer integrity, as demonstrated by decrease of TEER (Fig 8C). In addition, specific inhibition of iNKT cell–derived TNF and IFNγ abolished their pathogenic functions towards epithelial cell integrity (Fig 8D).

These findings suggest that exposure to mucosa-associated bacteria is sufficient to drive both innate and adaptive iNKT cell pro-inflammatory activation. Once activated, intestinal iNKT cells secrete pro-inflammatory cytokines conferring pathogenic features towards the intestinal epithelium.

### iNKT cell become functionally pro-inflammatory upon exposure to intestinal microbiota during intestinal inflammation in vivo

**Figure 9.**
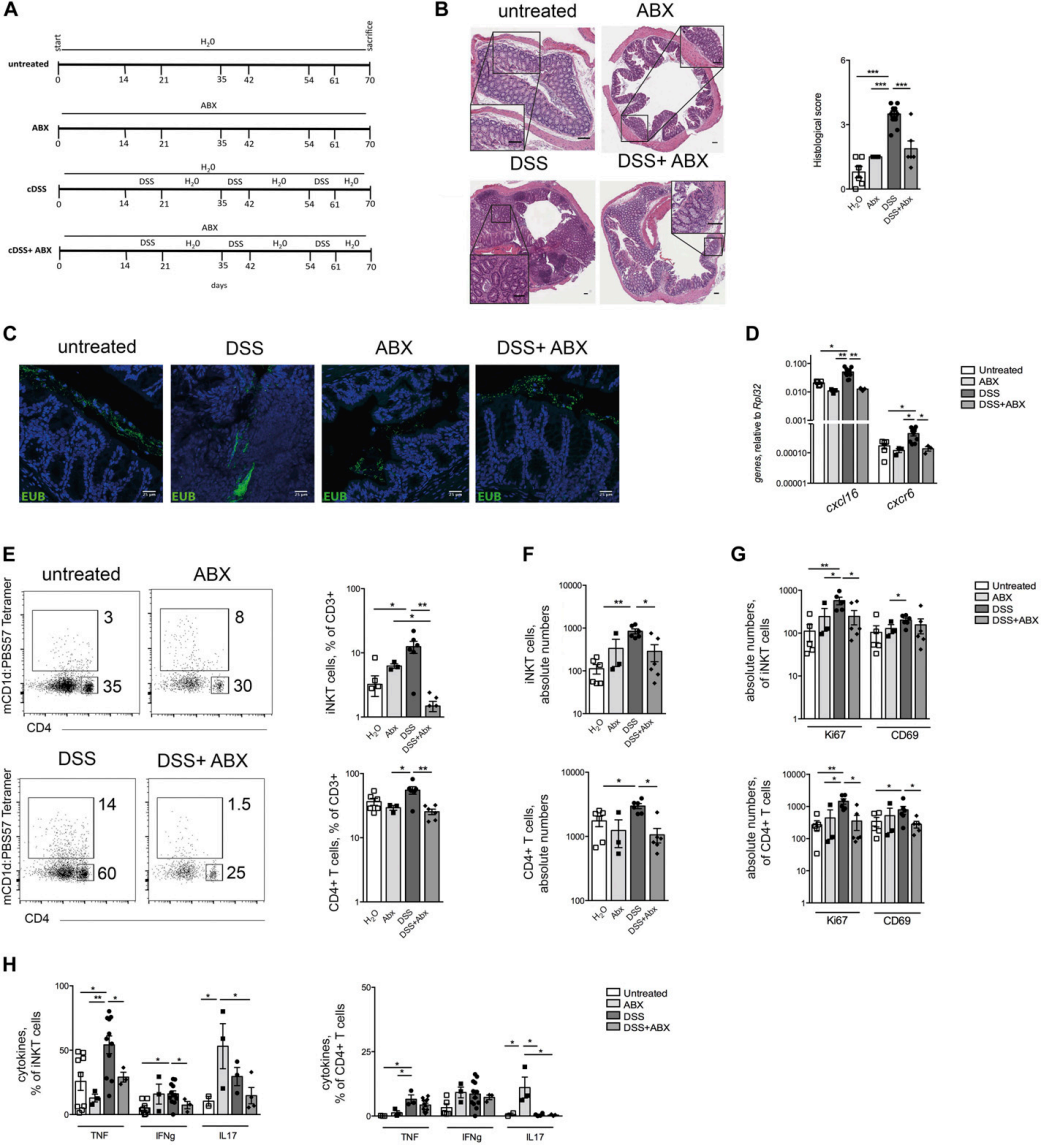
Intestinal microbiota elimination reduces pro-inflammatory activation of iNKT cells during experimental chronic colitis. **(A)** Schematic representation of the treatments. **(B)** H&E staining (scale bar: 100 μm) and cumulative histological score of colon specimens obtained from untreated (white bars), ABX-treated (light grey bars) DSS-treated (black bars), and DSS + ABX–treated (dark grey bars) mice. **(C)** Fluorescence microscopy of bacterial DNA after FISH with Eubacteria probes (Eub-488) in untreated, ABX-treated, DSS-treated, and DSS + ABX–treated mice as indicated. DAPI, staining of nuclei. **(D)** Colonic expression levels of *cxcl16* and *cxcr6* in mice untreated (white bars), ABX-treated (light grey bars), treated with DSS (black bars), or with DSS + ABX (dark grey bars). **(E)** Representative dot plots (left), frequency (right), and **(F)** absolute numbers of colonic iNKT cells and CD4^+^ T cells in mice untreated (white bars), ABX-treated (light grey bars), treated with DSS (black bars), or with DSS + ABX (dark grey bars). **(G)** Absolute number of ki67 and CD69 colonic iNKT cells (upper panels) and CD4^+^ T cells (lower panels) and **(H)** percentage of TNF-, IFNγ-, and IL17-producing iNKT cells (left panels) and CD4^+^ T cells (right panels) in mice untreated (white bars), ABX-treated (light grey bars), treated with DSS (black bars), or with DSS + ABX (dark grey bars). N = 4/5 group in two independent experiments. Statistical significance was calculated using the Kruskal–Wallis nonparametric test for multiple comparisons. *P* < 0.05 (*), *P* < 0.01 (**), *P* < 0.001 (***) were regarded as statistically significant. Error bars: mean ± SEM.

**Figure S12.**
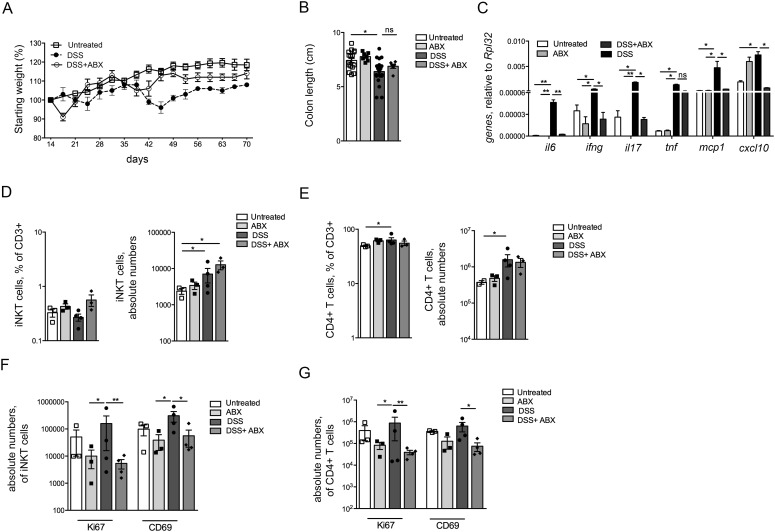
Intestinal microbiota elimination reduces pro-inflammatory activation of iNKT cells during experimental chronic colitis. **(A)** Weight loss in mice untreated (open squares), treated with DSS (closed circles), or with DSS + ABX (open circles). **(B, C)** Colon length (B) and colonic expression levels (C) of *il6, ifng, il17, tnf, mcp1*, and *cxcl10* in mice untreated (white bars), treated with ABX (light grey bars), DSS (black bars), or with DSS + ABX (dark grey bars). **(D, E)** Frequency (left panels) and absolute numbers (right panels) of iNKT cells (D) and of CD4^+^ T cells (E) in the mesenteric LNs of untreated (white bars), ABX-treated (light grey bars), DSS-treated (black bars), or DSS + ABX–treated (dark grey bars) mice. **(F, G)** Absolute numbers of iNKT cells (F) and of CD4^+^ T cells (G) in the mesenteric LNs of untreated (white bars), ABX-treated (light grey bars), DSS-treated (black bars) or DSS + ABX–treated (dark grey bars) mice. Statistical significant difference was assessed through one-way ANOVA with least significant difference post hoc test *P < 0.05, **P < 0.01.

Finally, we aimed at recapitulating in vivo the effects of bacteria exposure on pro-inflammatory activation of iNKT cells during intestinal inflammation. To this aim, chronic experimental intestinal inflammation was induced in mice by repetitive dextran sodium sulphate (DSS) administration in the presence or absence of broad-spectrum antibiotics (Figs 9A and S12). This treatment depleted the community of intestinal live bacteria, as demonstrated by fecal plating and CFU counting (data not shown). Epithelial barrier damage (Fig 9B) and signs of inflammation, including weight loss, colon shortening, and colonic expression of pro-inflammatory cytokines (Fig S12A–C), were strongly reduced by antibiotic administration (Hernandez-Chirlaque et al, 2016). Importantly, the antibiotic treatment greatly reduced the bacterial translocation (Fig 9C). In these mice, colonic expression levels of *CXCL16*, the chemokine responsible for iNKT cell chemoattraction into the gut (Geissmann et al, 2005; Olszak et al, 2014; Burrello et al, 2018a), and of its receptor *CXCR6* were strongly diminished (Fig 9D). Consistent with these observations, iNKT and CD4^+^ T cell frequencies (Fig 9E) and absolute numbers (Fig 9F) were decreased both in the colon and in the mesenteric LN (Fig S12D and E) of mice treated with DSS in the presence of antibiotics.

Most importantly, the reduction of intestinal bacterial translocation into the LP during intestinal inflammation significantly inhibited proliferation and CD69 up-regulation (Fig 9G), as well as pro-inflammatory cytokines secretion (Fig 9H) by both colonic (Fig 9G and H) and mesenteric LN-derived (Fig S12F and G) iNKT and CD4^+^ T cells.

In conclusion, these data collectively indicate that exposure to the intestinal microbiota is required for iNKT cell pro-inflammatory activation in the colonic mucosa and that iNKT and CD4^+^ T cells manifest in vivo similar pro-inflammatory activities during intestinal inflammation.

## Discussion

A functional involvement of iNKT cells has been suggested for a wide variety of human autoimmune disorders, including multiple sclerosis (Illes et al, 2000), rheumatic diseases (Van Der Vliet et al, 2001), and asthma (Akbari et al, 2006). Conversely, iNKT cell contribution to IBD pathogenesis is still incompletely understood. By studying human intestinal tissues, we showed that intestinal iNKT cells isolated from IBD patients possess a pro-inflammatory phenotype similar to that of conventional CD4^+^ T cells and manifest pathogenic features upon exposure to intestinal mucosa-associated microbiota. Thus, to our knowledge, this is the first comprehensive report of human intestinal iNKT cells in IBD patients.

Technical difficulties hampered for long time univocal attribution of iNKT cell pathogenic or protective roles in human diseases. Before the advent of the tetramer technology, (i) NKT cells could be identified either by the expression of NK-related markers (CD56 and/or CD161, the human counterpart of the murine NK1.1) together with CD3, by the reaction to CD1d stimulation, or by Vα24 TCR expression (Dellabona et al, 1994). It is believed, therefore, that old studies on iNKT cell functions most likely included different subsets, such as CD1d-restricted type 2 NKT, mucosal associated invariant T cells (MAIT), and γδ T cells (Mori et al, 2016). In addition, peripheral blood (PB) and tissue iNKT cell frequency is greatly variable among individuals (Chan et al, 2013). The analysis of the LPMC isolated from IBD patients and non-IBD donors confirmed this relevant intra-individual variability also for intestinal iNKT cell frequencies, which did not correlate with any epidemiologic or clinical parameters, including age, sex, concomitant therapy, disease localization, and duration.

Functionally different subsets of iNKT cells can be distinguished according to the expression of CD4 (Chan et al, 2013), whose engagement by CD1d molecules potentiates iNKT cell activation (Tonti et al, 2009). Human and murine tissue–derived iNKT cells are mostly CD4^+^ (Matsuda et al, 2008), and we here observed that up to 80% of human intestinal tetramer^+^ iNKT cells express CD4 both in HDs and IBD patients. CD161 expression, instead, has been specifically associated to intestinal tissue distribution of several T cell subsets, including MAIT cells and TCR γδ cells (Fergusson et al, 2011). In our study, LP iNKT cells from IBD patients and HDs mainly co-expressed CD4 and CD161 and secreted substantial amounts of pro-inflammatory cytokines. These data are in accordance with previous reports indicating that CD4^+^ CD161^+^ intestinal T cell subsets might exert specific pathogenic functions, both in CD (Cosmi et al, 2008) and UC patients (Fuss et al, 2004). Those reports, though, either excluded iNKT cells from the analysis (Cosmi et al, 2008) or focused on type II sulfatide-specific NKT subsets (Fuss et al, 2004; Fuss et al, 2014).

Here, we also report that both in mice and men, pro-inflammatory cytokine secretion is a key functional attribute of intestinal iNKT cells, leading to pathogenic activities against the intestinal epithelium.

Distinct Th subsets in the gut are known to secrete specific Th1 or Th2 cytokines, although context-dependent functional plasticity has been demonstrated for murine and human intestinal Th subsets (Huber et al, 2017). Similarly to murine (Lee et al, 2015) and PB-derived cells (Snyder-Cappione et al, 2010), in our analyses, intestinal iNKT cells possess an intrinsic capability to secrete a broad array of Th1, Th2, and Th17 cytokines. This characteristic, shared with other innate populations, could be an evolutionary conserved functional requirement for cells endowed with mucosal surfaces patrolling roles (Bamias et al, 2014). Indeed, similarly to other unconventional T cell subsets (Dunon et al, 1997), iNKT cells colonize mucosal tissues very early during ontogeny (Nieuwenhuis et al, 2009) as a consequence of an elevated epithelial expression of CXCL16 (Olszak et al, 2012).

Healthy and IBD-derived mucosa-associated microbiota were capable to efficiently activate in vitro human intestinal iNKT cells, stimulate cytokine secretion, and induce pathogenic functions. Several evidences suggest a reciprocal influence of iNKT cells and the intestinal gut microbiota (Nieuwenhuis et al, 2009; Wingender et al, 2012; Burrello et al, 2018b). iNKT cells affect murine gut colonization by commensal microorganisms (Nieuwenhuis et al, 2009), whereas during early neonatal and postnatal stages of development, commensal bacteria negatively shape iNKT cell repertoire (Olszak et al, 2012). The commensal *B. fragilis*, known to produce lipid antigens controlling homeostatic iNKT cell proliferation and activation (An et al, 2014), was detected only in two HDs and three colon CD-derived samples. Further studies are required to understand if reduction of *B. fragilis* in IBD patients might correlate with pro-inflammatory functional skewing of human intestinal iNKT cells.

IBD patients harbor significant variations in the intestinal microbiota composition as compared with non-IBD controls (Kaser et al, 2010), defined by an overall decrease of α-diversity and also by alterations of microbial taxa relative abundances, that is, specific increase in *Proteobacteria* (such as *adherent-invasive E. coli* and *Enterobacteriaceae* in CD) and decrease in *Firmicutes* (such as *F. prausnizii*) (Gevers et al, 2014). These variations were confirmed in our samples, including the decrease in the butyrate-producing *Roseburia* (Machiels et al, 2014), *Blautia* (Takahashi et al, 2016), and *Odoribacter* (Morgan et al, 2012) in IBD versus HDs, as well as the increase of *Erysipelotricheaceae* in UC patients (Pascal et al, 2017).

From a mechanistic point of view, our data suggest that the loss of the barrier integrity might be the critical event exposing iNKT cells to the mucosa-associated microbial ecology. Once in contact to the microbiota, as we showed, intestinal iNKT cells could be activated through TCR-dependent and TCR-independent mechanisms (Brigl et al, 2003), fuelling intestinal inflammation and contributing to propagate pathogenic activities towards the intestinal epithelium. Increased epithelial barrier permeability is considered a crucial event for IBD development, as a “leaky gut” is deemed responsible for the breakdown of intestinal tolerance through an increased translocation of bacterial and luminal antigens (Michielan & D'inca, 2015). Primary defects in epithelial junctional proteins, as JAM-A, are sufficient to increase translocation of bacteria in the LP (Laukoetter et al, 2007).

So far, few bacteria-derived glycoshphingolipid antigens capable to specifically activate iNKT cells have been identified, including those isolated from the cell wall of Gram-negative *Sphingomonas spp.*, *Borrelia burgdoferi*, and *Mycobacteria* (Fischer et al, 2004; Kinjo et al, 2005; Mattner et al, 2005; Sriram et al, 2005; Kinjo et al, 2006). Conversely, no iNKT-specific lipid antigens have been isolated from *adherent invasive E. coli* and *Salmonella*, two well-known intestinal pathobionts consistently present in our IBD- and non-IBD–derived samples and which efficiently stimulated iNKT cell responses partially through CD1d-dependent mechanisms. Abundant evidences instead exist that iNKT cells can be activated in an innate fashion by microbial products such as LPS (Brigl et al, 2003; Mattner et al, 2005), either directly through TLR4 binding (Askenase et al, 2005) or after IL-12/IL-18–mediated activation of LPS-stimulated dendritic cells (Nagarajan & Kronenberg, 2007). Our data suggest that in addition to an innate microbiota-dependent iNKT cell activation, endogenous lipid antigens might be induced or up-regulated in bacteria-stimulated moDC, thus explaining the observed antigen-specific activation by iNKT cells.

Upon exposure to the commensal intestinal microbiota, activated intestinal iNKT cells secreted a broad array of cytokines, including TNF and IFNγ, which are known to increase intestinal permeability (Vercammen et al, 2008), and those were directly responsible for in vitro iNKT cell pathogenic functions. We recently demonstrated that human Th17 cells isolated from the ileum of CD patients co-secrete pro-inflammatory IFNγ and TNF conferring pathogenic properties against the intestinal epithelium (Nizzoli et al, 2018), suggesting that during intestinal inflammation, iNKT cells and conventional CD4^+^ T cells might manifest a similar behavior.

Differently to conventional Th cells, though, iNKT cells can also behave as innate cells that, upon loss of barrier integrity, rapidly and massively respond to the commensal microbiota translocation into the LP. Abolishment of bacterial translocation by broad-spectrum antibiotic treatment during experimental chronic colitis efficiently blocked iNKT (and conventional CD4^+^ T cells) activation and cytokine secretion, recapitulating in vivo what observed in vitro and providing a rationale for possible targeted interventions aimed at containing immune cell responses in IBD patients.

In conclusion, our study sheds novel light on the pathogenic functions of iNKT cells during intestinal inflammation in IBD patients. Moreover, it suggests that a wider knowledge of the human microbiome at the community level, rather than on single microbial species, can better contribute to the final understanding of the microbiota role during human pathologies. Finally, this work indicates that during intestinal inflammation, iNKT cells share similar pro-inflammatory functions to conventional T cells, thus contributing to the fuelling of inflammatory processes.

## Materials and Methods

### Human subjects

Buffy-coated blood (HD, n = 15; IBD, n = 5) and intestinal specimens of UC patients (n = 16), CD patients (n = 24), and patients undergoing intestinal surgical resection for pathologies unrelated to IBD, including diverticular disease and intestinal tumors (n = 27) were obtained from the IRCCS Policlinico Ospedale Maggiore, Milan, Italy. The clinical characteristics and concomitant therapies of IBD patients are summarized in Table 1.

### Cells isolation

Human PBMCs were isolated by Ficoll-Hypaque gradient (Sigma-Aldrich). Human LPMCs were isolated as previously described (Caprioli et al, 2008). Briefly, the dissected intestinal mucosa was freed of mucus and epithelial cells in sequential steps with DTT (0.1 mmol/l) and EDTA (1 mmol/l) (both from Sigma-Aldrich) and then digested with collagenase D (400 U/ml) (Worthington Biochemical Corporation) for 5 h at 37°C. LPMCs were then separated with a Percoll gradient and cultured in complete RPMI 1640 medium containing 5% human serum (Sigma-Aldrich) and 100 U/ml IL-2 (Proleukin).

### Intestinal iNKT cell line and clone generation

Human iNKT cell lines were generated from sorted CD45^+^CD3^+^ CD1d:PBS57Tet^+^ cells isolated from total LPMCs or PBMCs.

Sorted iNKT cells were expanded in vitro for 2 wk in the presence of irradiated peripheral blood feeders, hIL2 (100 U/ml; Proleukin) and PHA (1 μg/ml; Sigma-Aldrich).

iNKT cell clones were generated via cloning by limiting dilution according to the protocol described in Libero (2009) and re-stimulated with irradiated feeder cells, PHA (1 μg/ml; Sigma-Aldrich) and hIL-2 (100 U/ml; Proleukin) every 21 d.

### iNKT-cell in vitro stimulation

In vitro polycolonal iNKT cell stimulation was performed with 0.1 μM PMA and 1 μg/ml ionomycin (Sigma-Aldrich) for 3 h. Neutralizing antibodies to CD1d (BD) were used at the concentration of 10 μg/ml.

For antigen-specific stimulation, 5 × 10^4^ antigen-presenting cells (moDC or CD45^+^HLA-DR^+^ intestinal LPMCs) were plated in each well in 1:1 ratio with human iNKT cells. Sonicated αGalCer was used at 40 ng/ml.

For bacteria stimulation assays, purified bacterial strains (*AIEC* LF82 and *S. typhimurium*) underwent cycles of heat inactivation and freezing/thawing before being serially diluted, starting from 5 × 10^6^ CFU/well (100:1 bacteria:APC) to 5 × 10^5^/well (1:1 bacteria:APC). The isolates of patient mucosa-associated microbiota were normalized according to their protein content (Pierce BCA Protein Assay kit; Thermo Fisher Scientific). After 36 h, T cell activation was estimated by measuring cytokine released in culture supernatants by ELISA assays, cytometric bead array (BD) or intracellular staining.

### Flow cytometry

Human and murine cells were stained with combinations of directly conjugated antibodies as specified in Table S3, all sourced from BD, eBioscience, or BioLegend. The gating strategy to identify human iNKT cells is described in Fig S1.

Table S3 Antibodies and FACS reagents.

Intracellular cytokines were detected after stimulation of human cells (iNKT cells and conventional T cells) for 3 h with 0.1 μM PMA and 1 μg/ml ionomycin (Sigma-Aldrich). 10 μg/ml Brefeldin A (Sigma-Aldrich) was added for the last hour of stimulation. The cells were fixed and permeabilized with Cytofix/Cytoperm (BD) before the addition of the antibodies, detecting the cytokine released.

Multiplexing analysis of cytokines in supernatants collected after in vitro stimulation was performed with a cytometric bead array assay, according to the manufacturer’s protocol (BD).

The samples were analyzed by a FACSCanto flow cytometer (BD), gated to exclude nonviable cells on the basis of light scatter. Data were analyzed using FlowJo software (Tristar).

### Microbiota identification by 16S rRNA gene amplification, sequencing, and data analysis

Faeces and mucus scraped from the colon were stored at −80°C until the DNA was extracted with GNOME DNA Isolation Kit (MP) following the protocol described in the study by Furet et al (2009). Partial 16S rRNA gene sequences were amplified using primer pair Probio_Uni and /Probio_Rev, targeting the V3 region of the 16S rRNA gene sequence (Milani et al, 2013b). 16S rRNA gene sequencing was performed using a MiSeq (Illumina) at the DNA sequencing facility of GenProbio srl (www.genprobio.com) according to the protocol previously reported (Milani et al, 2013a). Following sequencing, the obtained individual sequence reads were filtered by the Illumina software to remove low-quality and polyclonal sequences. All Illumina quality-approved, trimmed, and filtered data were exported as .fastq files. The .fastq files were processed using a custom script based on the QIIME software suite (Caporaso et al, 2010). Quality control retained sequences with a length between 140 and 400 bp and mean sequence quality score >20, whereas sequences with homopolymers >7 bp and mismatched primers were omitted. To calculate downstream diversity measures (α and β diversity indices, Unifrac analysis), 16S rRNA operational taxonomic units (OTUs) were defined at ≥99% sequence homology using uclust (Edgar, 2010) and OTUs with less than 10 sequences were filtered. All reads were classified to the lowest possible taxonomic rank using QIIME (Caporaso et al, 2010) and a reference dataset from the SILVA database. Biodiversity of the samples (α diversity) were calculated with Chao1 and Shannon indices. Similarities between samples (β diversity) were calculated by unweighted uniFrac (Lozupone & Knight, 2005). The range of similarities is calculated between the values 0 and 1. PCoA representations of β diversity were performed using QIIME (Caporaso et al, 2010).

### Measurement of TEER

The Caco-2 cells were sourced from the American Type Culture Collection and cultured in DMEM supplemented with 20% FCS, 2 mM L-glutamine, 1 mM sodium pyruvate, 0.1 mM nonessential amino acids and penicillin/streptomycin. The cells were split three times a week. For TEER measurement, the cells at passage 10–30 were plated at 15 × 10^3^ cells/well on polyester permeable Transwell-Clear inserts (6.5-mm diameter, 0.4-mm pore size; Corning) and grown for 5−7 d, until ΔTEER >300 Ω*cm2 (Millicell-ERS Volt-Ohm Meter; Millipore).

The supernatants collected from intestinal CD4^+^ T cell clones, stimulated for 3 h with PMA/ionomycin in Caco-2 medium, in the presence or absence of neutralizing Ab (anti-human -IFNγ, -IL-17A, -TNF [eBioscience], -IL13 [BioLegend] at a concentration of 20 μg/ml for anti-IFNγ and 10 μg/ml for the others) were applied in the lower Transwell chamber.

Measurements were carried out every 30 min in the first 2 h, then at 24 and 48 h after stimulation. The ohmic resistance of a blank (culture insert without cells) was measured in parallel. To obtain the sample resistance, the blank value was subtracted from the total resistance of the sample. The final unit area resistance (Ω*cm2) was calculated by multiplying the sample resistance by the effective area of the membrane. For comparison among treatments with different clones, TEER was normalized to the supernatant of each unstimulated clone.

### Mice and induction of murine experimental intestinal inflammation

C57BL/6 mice (Charles River, IT) of 8–10 wk of age were housed at the European Institute of Oncology (IEO) animal facility in SPF conditions. Littermates of the same sex and age were randomly assigned to the different experimental groups.

For the induction of DSS-induced chronic colitis, the mice were given 2% (wt/vol) DSS (MW 40 kD; TdB Consultancy) in their drinking water for 7 d followed by 2 d of recovery.

To eliminate the gut microflora, the mice were administered with a mix of neomycin (1g/l), ampicillin (1g/l), vancomycin (0.5 g/l), and metronidazole (1g/l) in their drinking water throughout the DSS treatment as described above. The weight curve was determined by weighing the mice daily. At euthanization, the colons were collected, their length was measured and divided in portions to be fixed in 10% formalin for histological analyses, and they were snap-frozen for RNA extraction and for LPMC immunophenotyping.

### Histological analysis

Tissue processing was performed with a LEICA PELORIS processor before paraffin embedding. Murine samples were included using an automated system (SAKURA Tissue-Tek) as previously described (Nizzoli et al, 2018). After hematoxylin and eosin staining, snapshots of histology were taken using an Aperio CS2 microscope with a scanning resolution of 50,000 pixels per inch (0.5 μm per pixel with 10× objective and 2.5 μm per pixel when scanning at 4×). Scoring of disease activity was performed according to the criteria described in Table S4.

Table S4 Scoring scheme for the evaluation of intestinal inflammation.

### FISH

Formalin-fixed paraffin-embedded tissues were sectioned to 5 μm thickness. The probes (EUB1, EUB2, and EUB3) used were designed to specifically target different regions of the 16S rRNA. All the probes were manufactured by Sigma-Aldrich and labelled with Alexa488. Probes were applied to slides at a concentration of 5 ng/μl in prewarmed hybridization buffer (0.9 M NaCl, 20 mM Tris, pH 7.4, and 0.01% SDS). The slides were incubated overnight at 50°C in a humid chamber and washed at 50°C in pre-warmed washing buffer (0.9 M NaCl and 20 mM Tris, pH 7.4). The slides were counterstained with DAPI. Confocal images were acquired through HCX PL APO 40X (NA 1.25) oil immersion objective. The probes sequences are listed in Table S5.

Table S5 FISH probes.

### RNA isolation, cDNA synthesis, quantitative PCR, and gene expression

Total RNA from intestinal tissues was isolated using TRIZOL and Quick-RNA MiniPrep (ZymoResearch) according to the manufacturer’s specifications and following the *MetaHIT* project guidelines. cDNAs were generated from 1 μg of total RNA with reverse transcription kit (Promega). Gene expression levels were evaluated by qPCR and normalized to RpL32 gene expression. The primer sequences are listed in Table S6.

Table S6 Primer sequences (mouse).

### Murine cell isolation

Peyer’s Patches were removed, and colonic LPMCs were isolated via incubation with 5 mM EDTA at 37°C for 30 min, followed by further digestion with collagenase IV and DNase at 37°C for 1 h. The cells were then separated with a Percoll gradient. In some experiments after isolation, the cells were re-stimulated in vitro for 3 h with PMA/ionomycin in the presence of Brefeldin A for cytokine secretion.

### Statistics

Statistical significance was calculated using a Wilcoxon matched-pairs signed-rank *t* test not parametric and not assuming Gaussian distribution. *P* < 0.05 (*), *P* < 0.005 (**), and *P* < 0.0005 (***) were regarded as statistically significant.

### Study approval

The Institutional Review Board approved the study (permission ref. no. EA1/107/10), and informed consent was obtained from the subjects involved in the study. Animal procedures were approved by Italy’s Ministry of Health (Authorizations no. 127/15, 27/13, 913/16, 415/17).

## Supplementary Material

Reviewer comments
