# Supplementary figures and images for: Mucosa-associated microbiota drives pathogenic functions in IBD-derived intestinal iNKT cells
Source: Life Sci Alliance. 2019 Feb 13;2(1):e201800229. doi: 10.26508/lsa.201800229 (PMC6374994; doi:10.26508/lsa.201800229)

**Table S1: Statistical summary of iNKT cell cytokine production.**


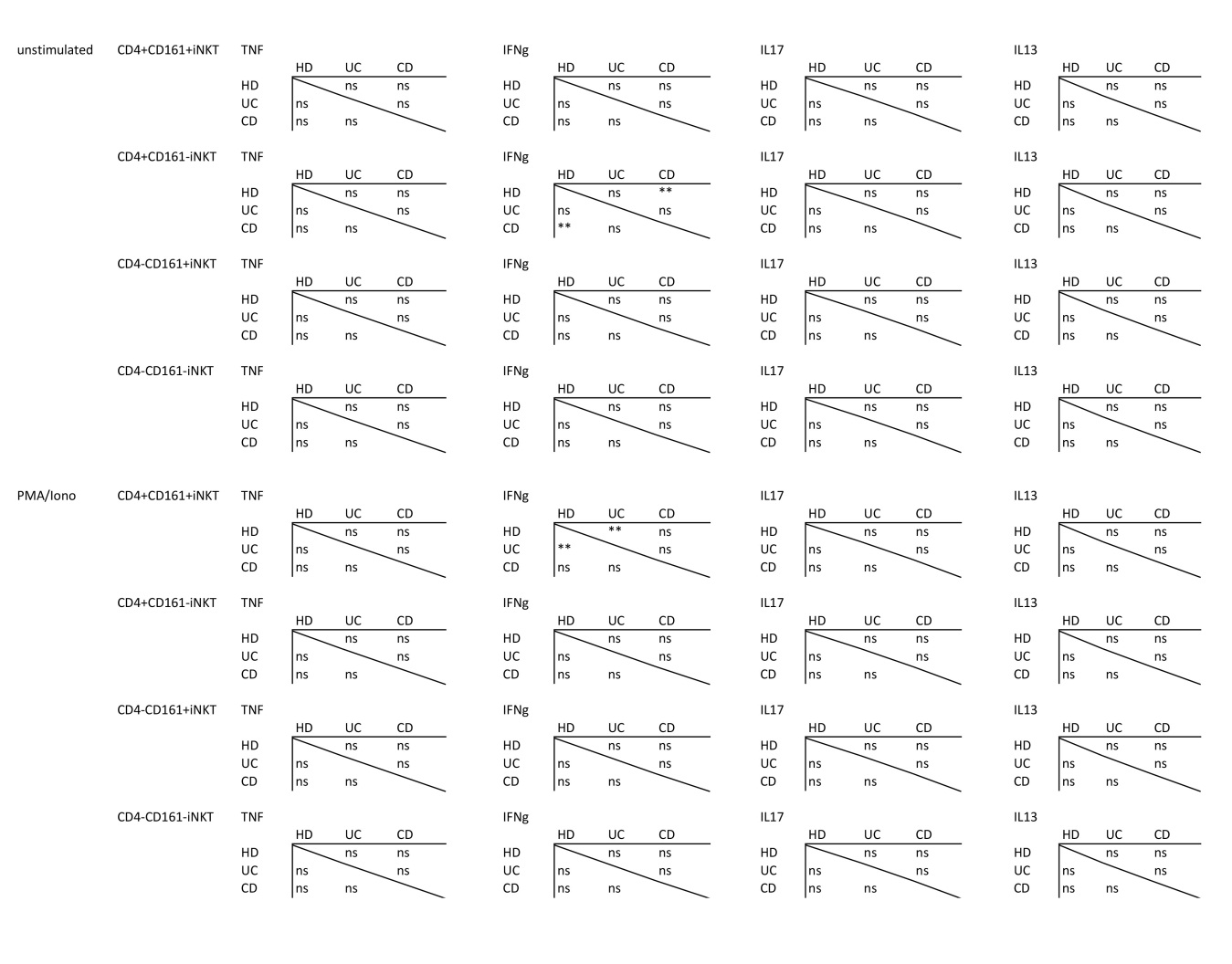

Supplement: Supplementary file 1 [file LSA-2018-00229_TableS1.docx]

**Table S2: Statistical summary of conventional T cell cytokine production.**


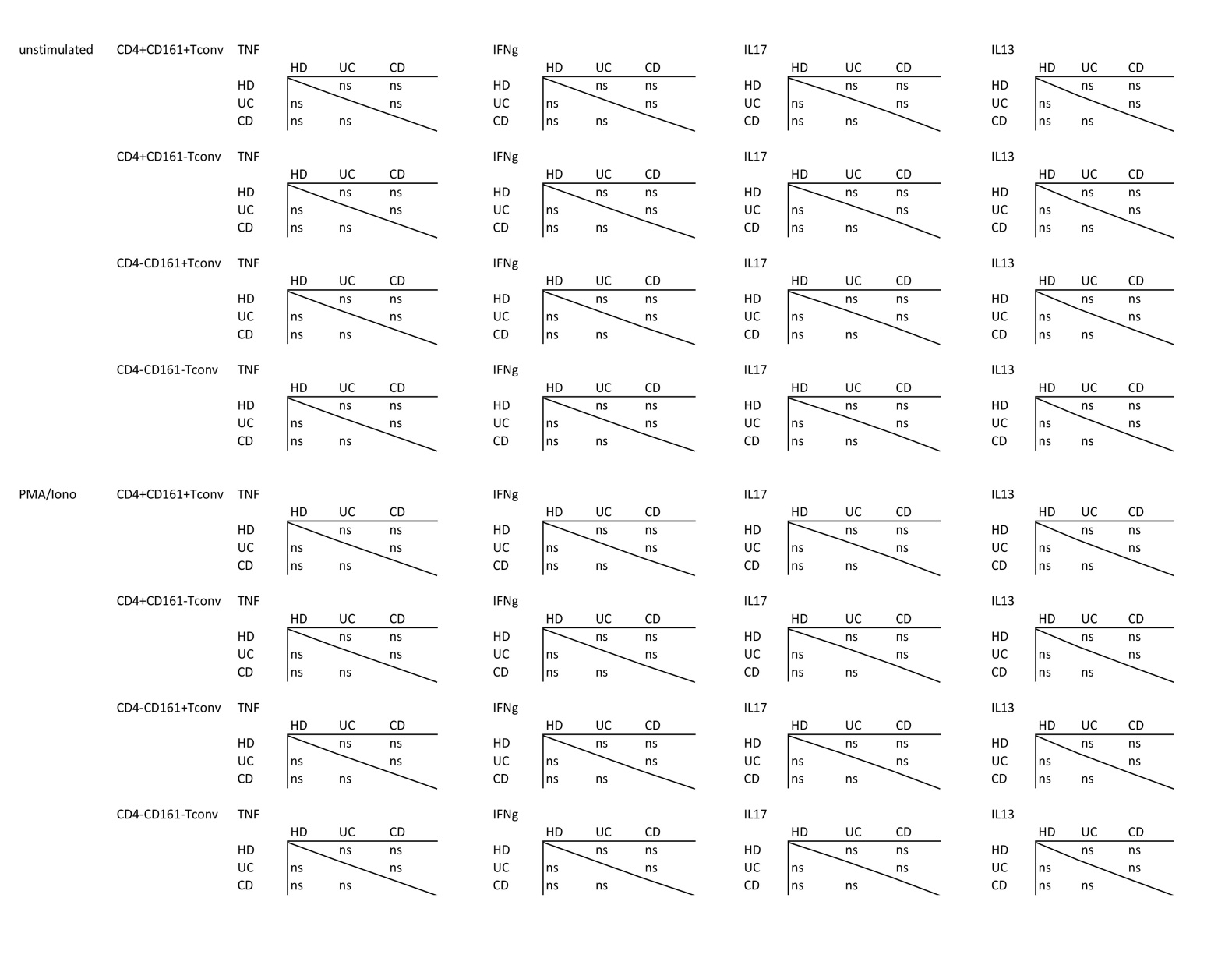

Supplement: Supplementary file 2 [file LSA-2018-00229_TableS2.docx]
