# Supplementary material for: Mucosa-associated microbiota drives pathogenic functions in IBD-derived intestinal iNKT cells
Source: Life Sci Alliance. 2019 Feb 13;2(1):e201800229. doi: 10.26508/lsa.201800229 (PMC6374994; doi:10.26508/lsa.201800229)
Supplement: Supplementary file 3 [file LSA-2018-00229_TableS3.docx]

**Table S3: Antibodies and FACS reagents.**

| **Human Antibodies** | **clone** | **Vendor** |
| --- | --- | --- |
| hCD1d:PBS57 Tet |  | Gift from NIH Tet facility |
| CD3 | OKT3 | Tonbo |
| CD4 | RPA-T4 | Tonbo |
| CD8a | OKT8 | Tonbo |
| CD25 | BC96 | BD |
| CD11c | B-ly6 | BD |
| CD19 | HIB19 | BD |
| IL17A | BL168 | Biolegend |
| IFNg | B27 | BD |
| TNF | Mab11 | BD |
| IL13 | 142928 | R&D |
| Zombie Dye | Cat #423104 | Biolegend |
| Anti-CD1d | CD1d42 | BD |
| Anti-IFNg | NIB42 | eBioscience |
| Anti-TNF | Mab1 | eBioscience |
| Anti-IL17 | eBio64CAP17 | eBioscience |
| Anti-IL13 | JES10-5A8 | Biolegend |
|  |  |  |
| **Mouse Antibodies** | **clone** | **Vendor** |
| mCD1d:PBS57Tet |  | Gift from NIH Tet facility |
| CD45.2 | 104 | Biolegend |
| CD3 | 17A2 | BD |
| CD8a | 53-6.7 | eBioscience |
| CD4 | GK1.5 | BD |
| CD11c | HL3 | BD |
| CD19 | 1D3 | BD |
| CD11b | M1/70 | BD |
| CD69 | H1.2F3 | BD |
| Ki67 | 16A8 | Biolegend |
| TNF | MP6-XT22 | Biolegend |
| IFNg | XMG1.2 | BD |
| IL17 | TC11-18H10.1 | Biolegend |
|  |  |  |
|  |  |  |
|  |  |  |
| **Human CBA** | **code** | **Vendor** |
| IFNg | 560111 | BD |
| TNF | 558273 | BD |
| IL17A | 560383 | BD |
| IL13 | 558450 | BD |
|  |  |  |
|  |  |  |
