# Supplementary material for: Mucosa-associated microbiota drives pathogenic functions in IBD-derived intestinal iNKT cells
Source: Life Sci Alliance. 2019 Feb 13;2(1):e201800229. doi: 10.26508/lsa.201800229 (PMC6374994; doi:10.26508/lsa.201800229)
Supplement: Supplementary file 4 [file LSA-2018-00229_TableS4.docx]

**Table S4: Scoring scheme for the evaluation of intestinal inflammation.**

|  | **Criterion** | **Definition** | **Score value** |
| --- | --- | --- | --- |
| **Inflammatory cell infiltrate** | Severity (leukocyte density of lamina propria area infiltrated in evaluated hpf) | No infiltrate | 0 |
|  |  | Minimal acute (<10%) | 0.25 |
|  |  | Mild chronic (10-25%, scattered neutrophils) | 0.5 |
|  |  | Moderate chronic (26-50%) | 0.75 |
|  |  | Marked (>51%, dense infiltrate) | 1 |
|  | Extent (expansion of leukocyte infiltration) | Mucosal | 0.5 |
|  |  | Mucosal and submucosal | 0.75 |
| **Epithelial changes** | Hyperplasia (increase in epithelial cell numbers in longitudinal crypts, visible as crypt elongation) | No hyperplasia | 0 |
|  |  | Minimal (<25%) | 0.25 |
|  |  | Mild (26-35%) | 0.5 |
|  |  | Moderate (36-50%, mitoses in the upper third of the crypt epithelium) | 0.75 |
|  |  | Marked (>51%, mitoses in crypt epithelium distant from crypt base) | 1 |
|  | Goblet cell loss (reduction of goblet cell numbers relative to baseline goblet cell numbers per crypt) | No loss | 0 |
|  |  | Minimal (<25%) | 0.25 |
|  |  | Mild (26-35%) | 0.5 |
|  |  | Moderate (36-50%) | 0.75 |
|  |  | Marked (>51%) | 1 |
| **Mucosal architecture** | Ulceration (epithelial defect reaching beyond muscolaris mucosae) | No ulcers | 0 |
|  |  | Ulcers | 0.25 |
|  | Granulation tissue (connective tissue repair with new capillaries, surrounded by infiltrating cells, hypertrophied areas) | No granulation tissue | 0 |
|  |  | Granulation tissue | 0.25 |
|  | Mucosal thickness and crypt depth | No thickening | 0 |
|  |  | Thickening | 0.5 |
|  | Glandular rarefaction | No rarefaction | 0 |
|  |  | Rarefaction | 0.5 |
|  | Dysplasia | No dysplasia | 0 |
|  |  | Dysplasia | 0.5 |
|  |  | **MAX SCORE** | **6** |
