# Supplementary material for: Mucosa-associated microbiota drives pathogenic functions in IBD-derived intestinal iNKT cells
Source: Life Sci Alliance. 2019 Feb 13;2(1):e201800229. doi: 10.26508/lsa.201800229 (PMC6374994; doi:10.26508/lsa.201800229)
Supplement: Supplementary file 5 [file LSA-2018-00229_TableS5.docx]

**Table S5: FISH probes.**

| **FISH Probes** | **clone** | **Vendor** |
| --- | --- | --- |
| EUB1 | 5’(A488)-GCTGCCTCCCGTAGGA | SIGMA |
| EUB2 | 5’(488)-GCAGCCACCCGTAGGTG | SIGMA |
| EUB3 | 5’(488)-GCTGCCACCCGTAGGTG | SIGMA |
| NON –EUB Scramble | 5’(Cyanine5)-CGACGGAGGGCATCCTCA | SIGMA |
