# Supplementary material for: Mucosa-associated microbiota drives pathogenic functions in IBD-derived intestinal iNKT cells
Source: Life Sci Alliance. 2019 Feb 13;2(1):e201800229. doi: 10.26508/lsa.201800229 (PMC6374994; doi:10.26508/lsa.201800229)
Supplement: Supplementary file 6 [file LSA-2018-00229_TableS6.docx]

**Table S6: Primer sequences (mouse).**

| **Primer** | **Product size (bp)** | | | **Vendor** |
| --- | --- | --- | --- | --- |
| IL17 | 94 | | | Qiagen (QuantiTect) |
| IFNg | 190 | | | Qiagen (QuantiTect) |
| Rpl32 | 117 | | | Qiagen (QuantiTect) |
|  |  | | |  |
|  |  | | |  |
| **Primer** | **Forward** | **Reverse** | **bp** | **Vendor** |
| CXCL16 | AGCGCAAAGAGTGTGGA | GGTTGGGTGTGCTCT | 193 | SIGMA |
| CXCR6 | CCTTTTTGGGCCTATGCA | ATGCCTCGAAGAGTT | 71 | SIGMA |
| MCP-1 | CAAGATGATCCCAATGA | GGTTCCGATCCAGGT | 161 | SIGMA |
| CXCL10 | CGCTGCAACTGCCATCCA | CCGGATTCAGACATC | 148 | SIGMA |
| TNF | TCTTCTCATTCCTGCTTG | CACTTGGTGGTTTGCT | 200 | SIGMA |
| IL6 | CTCTGGGAAATCGTGGA | GCAAGTGCATCATCG | 77 | SIGMA |
