# Supplementary material for: Mucosa-associated microbiota drives pathogenic functions in IBD-derived intestinal iNKT cells
Source: Life Sci Alliance. 2019 Feb 13;2(1):e201800229. doi: 10.26508/lsa.201800229 (PMC6374994; doi:10.26508/lsa.201800229)
Supplement: Reviewer comments [file LSA-2018-00229_review_history.pdf]

Mucosa-associated microbiota drives pathogenic functions in IBD-derived intestinal iNKT cells

Claudia Burrello, Gabriella Pellegrino, Maria Rita Giuffrè, Giulia Lovati, Ilaria Magagna, Alice Bertocchi, Fulvia Milena Cribiù, Francesca Boggio, Fiorenzo Botti, Elena Trombetta, Laura Porretti, Antonio Di Sabatino, Maurizio Vecchi, Maria Rescigno, Flavio Caprioli, Federica Facciotti
DOI: 10.26508/lsa.201800229

Review timeline:

Submission Date:	30 October 2018
Editorial Decision:	30 October 2018
Revision Received:	9 January 2019
Editorial Decision:	15 January 2019
Revision Received:	24 January 2019
Editorial Decision:	28 January 2019
Revision Received:	1 February 2019
Accepted:	1 February 2019

Report:

(Note: Letters and reports are not edited. The original formatting of letters and referee reports may not be reflected in this compilation.)

No full Peer Review Process File is available with this article, as the authors have chosen not to make the review process public in this case and as the original reports were obtained at another journal.

1st Editorial Decision 30 October 2018

Thank you for transferring your manuscript entitled "Mucosa-associated microbiota drives pathogenic functions in IBD-derived intestinal iNKT cells" to Life Science Alliance. The manuscript was assessed by expert reviewers at another journal before, and the editor transferred those review reports to us with your permission.

The reviewers who evaluated your study elsewhere thought that it remains rather unclear whether targeting iNKT cells in IBD patients may have therapeutic potential. They further noted that the causality currently proposed is not supported by the data provided. Based on the reviews already at hand, and as pre-discussed with you via phone and email, we would like to invite you to submit a revised version for publication in Life Science Alliance. We would expect re-writing of the text, toning down the causality currently proposed, and providing the requested statistical analyses. When revising your manuscript, please also check carefully that it is always clear in the figures (and the text) which cell lines you are using/referring to. Reviewer #2 also requested that you compare the proportion (and absolute number if possible) of cells across the groups. We understand that this is not feasible given that you work with surgical specimens and not whole organs and we appreciate the way you already proposed in a preliminary point-by-point response how to address this point by giving the relative numbers observed per assay.

To upload the revised version of your manuscript, please log in to your account:
https://lsa.msubmit.net/cgi-bin/main.plex
You will be guided to complete the submission of your revised manuscript and to fill in all necessary information.

We would be happy to discuss the individual revision points further with you should this be helpful.

While you are revising your manuscript, please also attend to the below editorial points to help expedite the publication of your manuscript. Please direct any editorial questions to the journal office.

The typical timeframe for revisions is three months.

We hope that the comments below will prove constructive as your work progresses.

Thank you for this interesting contribution to Life Science Alliance. We are looking forward to receiving your revised manuscript.

2nd Editorial Decision 15 January 2019

Thank you for submitting your revised manuscript entitled "Mucosa-associated microbiota drives pathogenic functions in IBD-derived intestinal iNKT cells" to Life Science Alliance.

We were not able to contact the original reviewers of your work (who reviewed this work at a different journal), and therefore sought technical advice on the revision performed (new parts in figures 1, 2, S1, S6, S7 and S10). We are afraid, based on the input received from the advisor, a further revision is required to make your work suitable for publication here. We would thus like to invite you to provide a further revised version, addressing the following points made by the advisor:

- The way the data are displayed is not appropriate: in all figures, the individual values should be plotted as dots following the recommendation in Weissgerber TL, Milic NM, Winham SJ, Garovic VD (2015) Beyond Bar and Line Graphs: Time for a New Data Presentation Paradigm. PLoS Biol 13(4): e1002128. doi:10.1371/journal.pbio.1002128.
- Figure 2, the number of cells studied for the Tet+ subset is probably very small (see comment on Fig. S1). It is unclear how experiment S1 was performed: no stimulation or stimulation with pma+I for how long, Any golgi inhibitor? How brief was the stimulation? Ionomycin PMA stimulation induces a rapid (6H) downregulation of CD4, how is this taken into account. Actual staining for the different cytokines on ex vivo or stimulated tet+ and Tet- Lamina propria CD4 T cells should get provided, this is not shown in figure S1.
- Figure S1, One panel is missing: it seems that only the CD3+TETneg cells are shown. I don't see the example on Tet+. Actually, the number of cells is probably very small and thus the data not very convincing.
- Figure S6, I am not sure that this figure is representative of all the cell lines and clones. It is surprising that the expression profile of the different markers is homogenous as shown in fig. S5. Statistics in the different pathological groups for CD137, CD69, CD4 and CD161 should be shown.
- Figure S7 The origin (healthy, UC, or CD) should be stated for the clones not only for the cell lines but also for the clones. Histograms should not be used and dot plots (with different dots for the different clones and different symbols for the pathological groups) should be used
- Figure S10: satisfactory

Thank you for this interesting contribution to Life Science Alliance. We are looking forward to receiving your revised manuscript.

3rd Editorial Decision 28 January 2019

Thank you for submitting your revised manuscript entitled "Mucosa-associated microbiota drives pathogenic functions in IBD-derived intestinal iNKT cells". We appreciate the introduced changes and we would be happy to publish your paper in Life Science Alliance pending final revisions necessary to meet our formatting guidelines:

- please note that some of the dot blots are not easily readable (dark grey bars as background), please modify
- please mention in your figure legends what kind of error bars are depicted in the graphs
- please consistently call-out your figure panels (either mention all individual panels or none): S Fig 5B is called out in the manuscript text, but not S Fig 5A; S Fig 7D is called out, but does not exist; S Fig 9A is called out, but not S Fig 9B
- you call out Figure 2D, please correct to Figure 2B
- please add a callout to the S tables
- please upload all files as individual figure files, including the supplementary figures. The legends

of the supplementary figures can get included in the manuscript text file, the supplementary tables can get added to this docx file, too
- please link your profile in our submission system to your ORCID iD, you should have received an email with instructions on how to do so

Thank you for this interesting contribution, we look forward to publishing your paper in Life Science Alliance.
